# Supplementary figures and images for: Diabetes regulates fructose absorption through thioredoxin-interacting protein
Source: eLife. 2016 Oct 11;5:e18313. doi: 10.7554/eLife.18313 (PMC5059142; doi:10.7554/eLife.18313)

**Figure 1 source data 1**


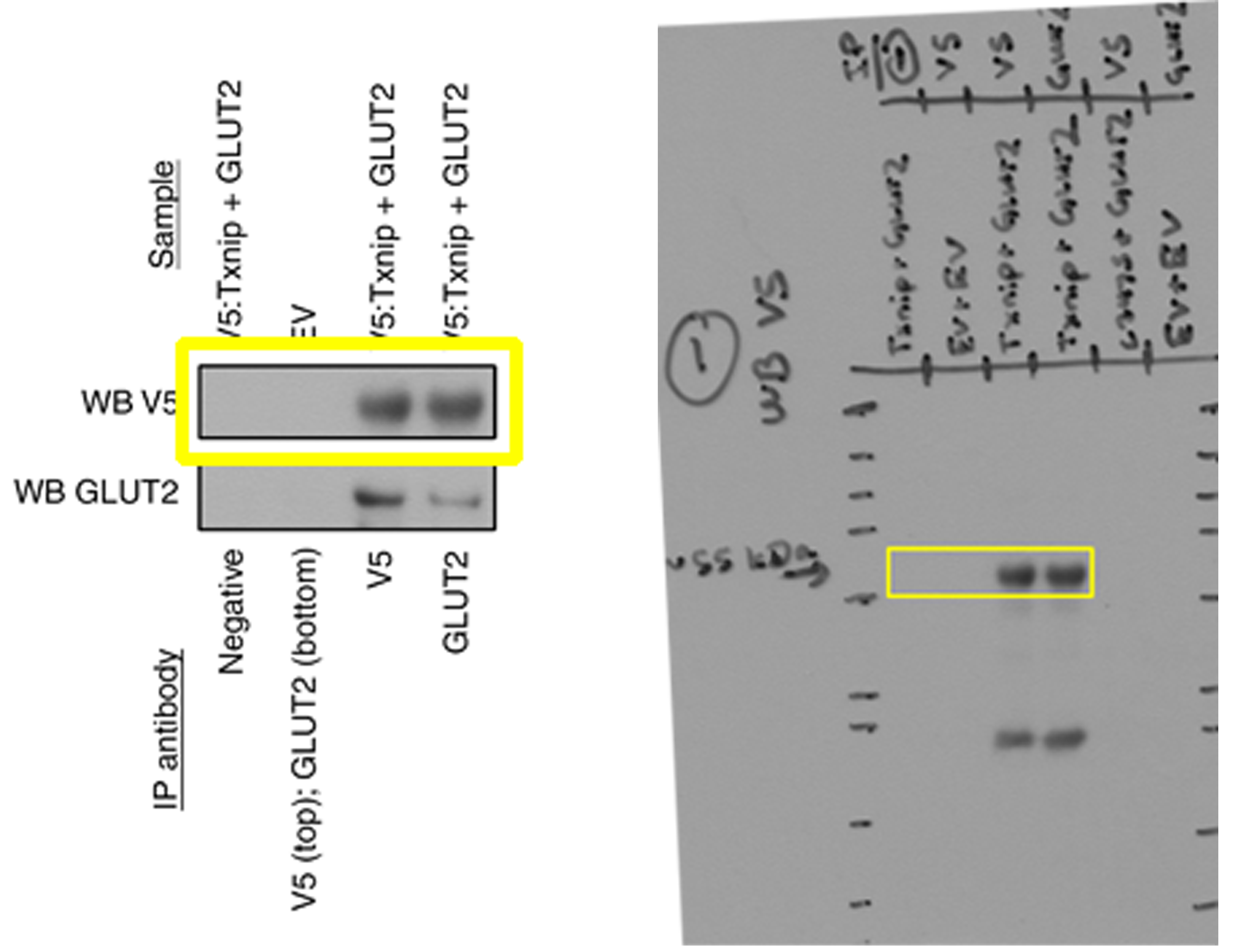

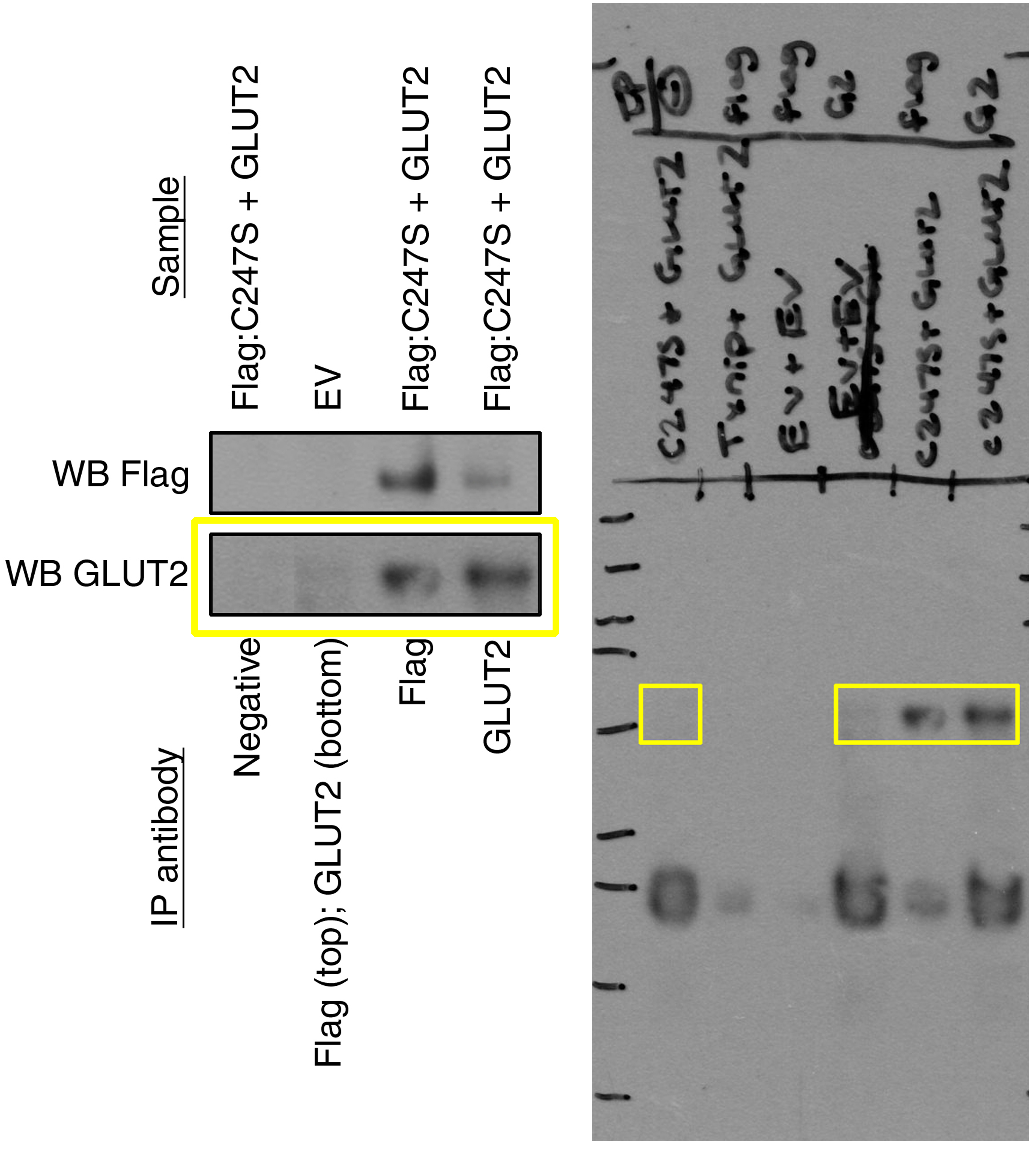


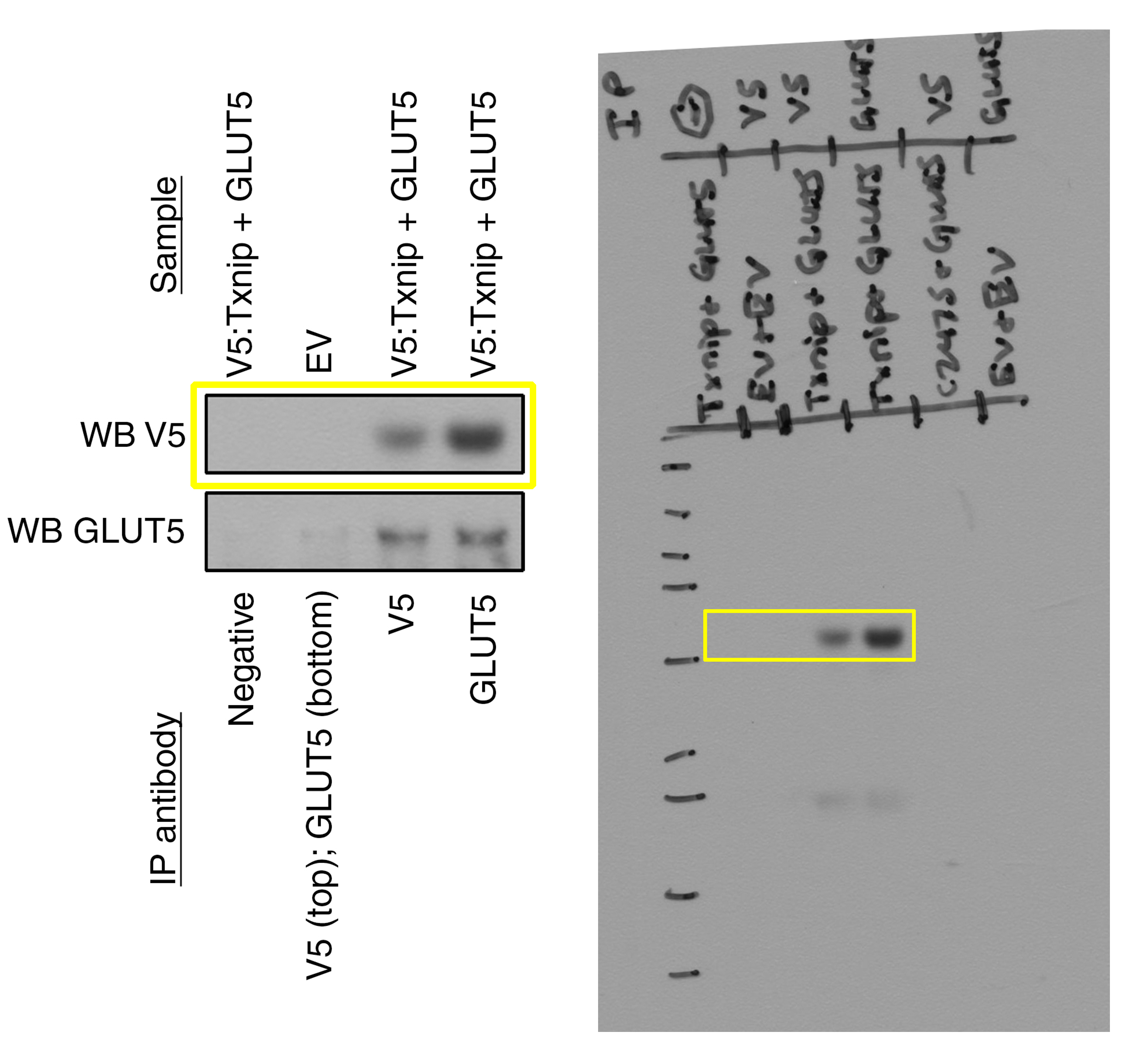

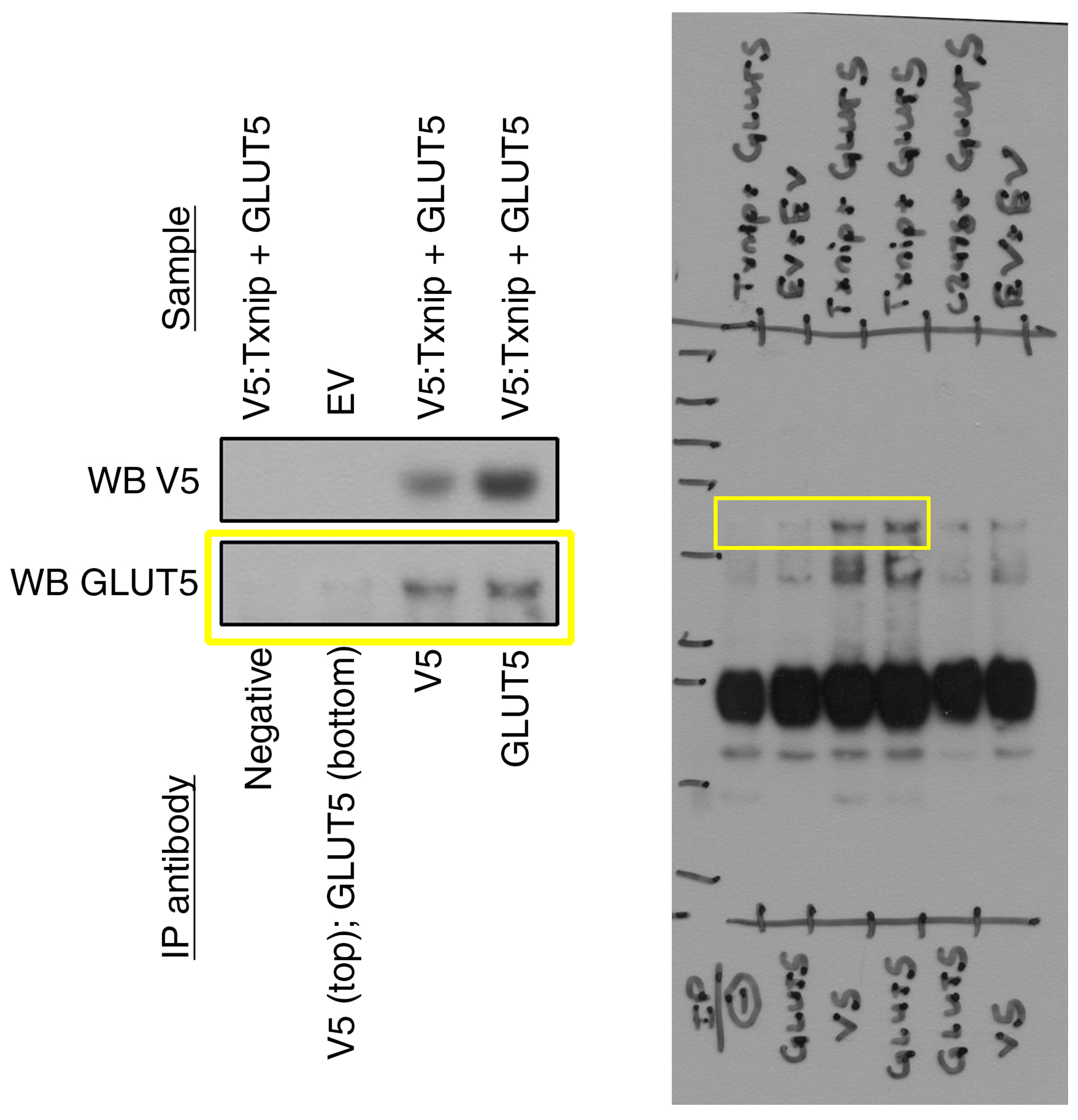


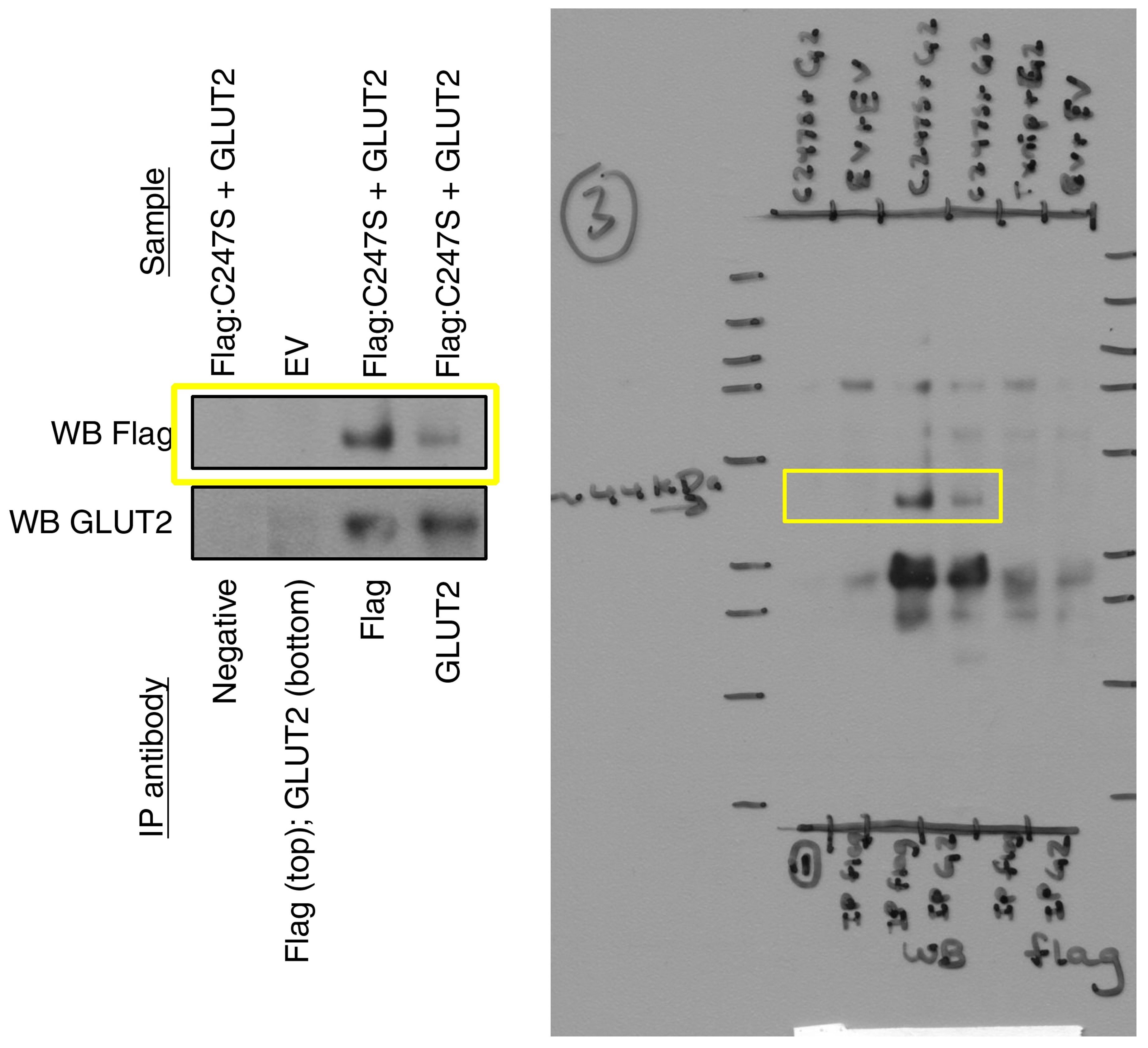

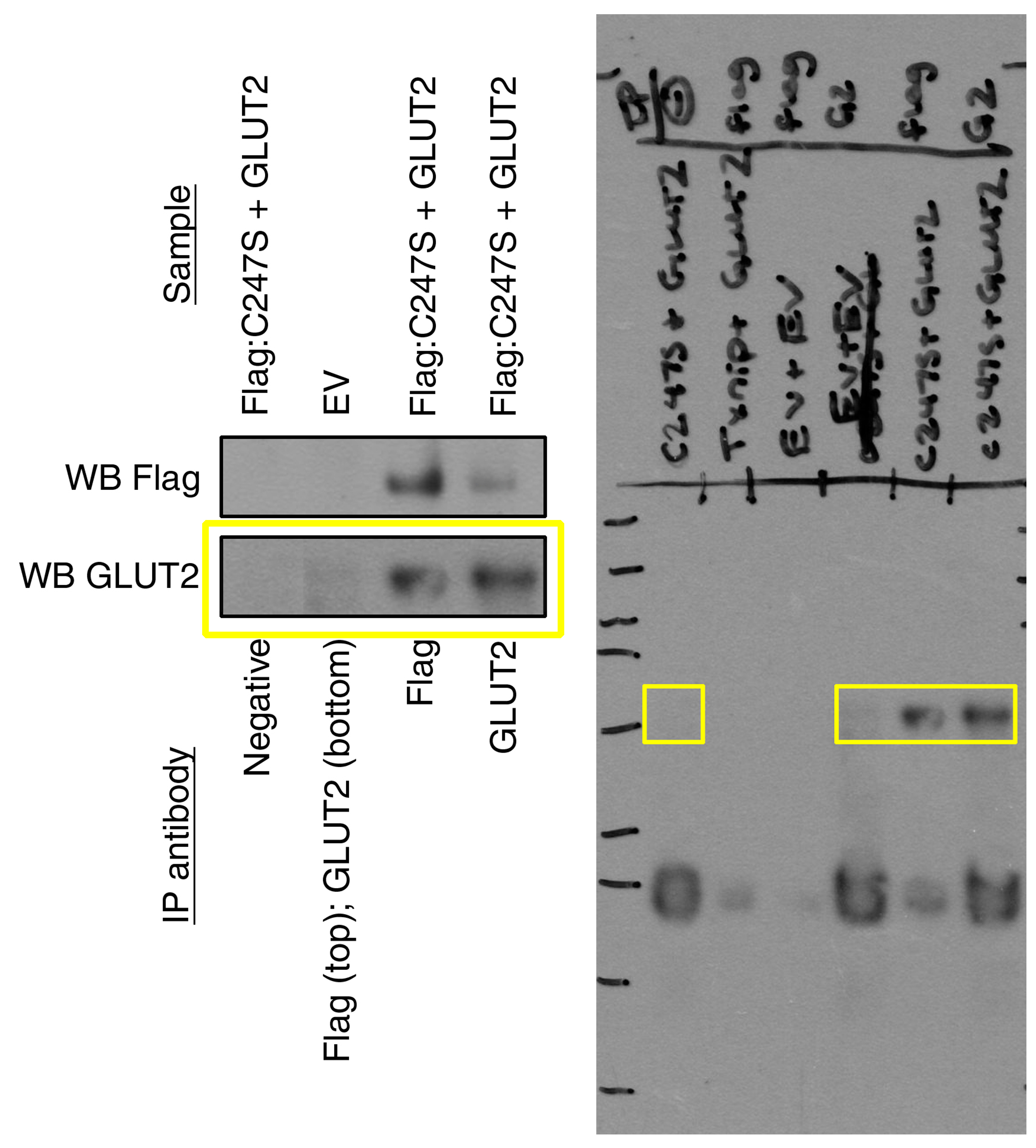


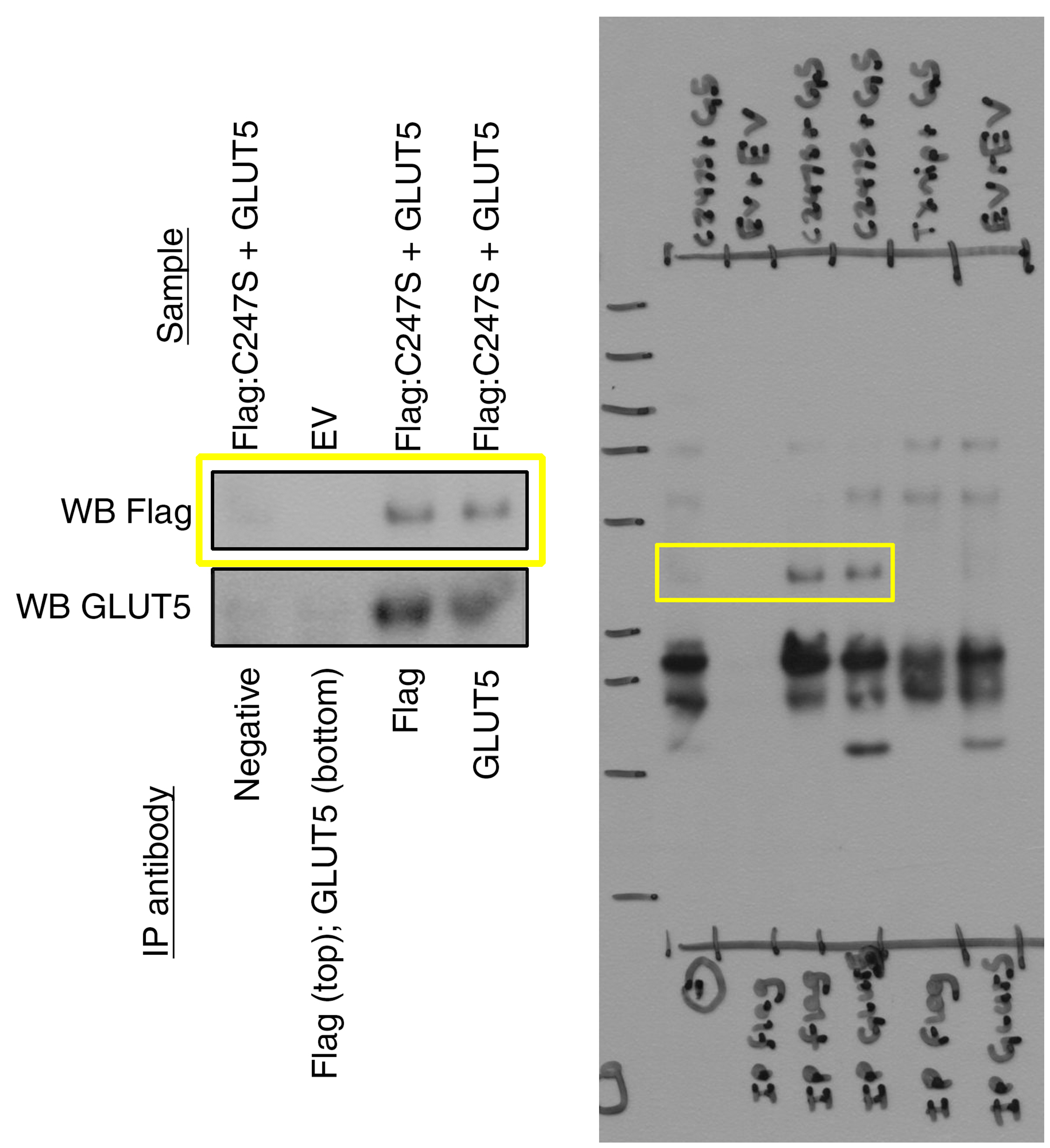

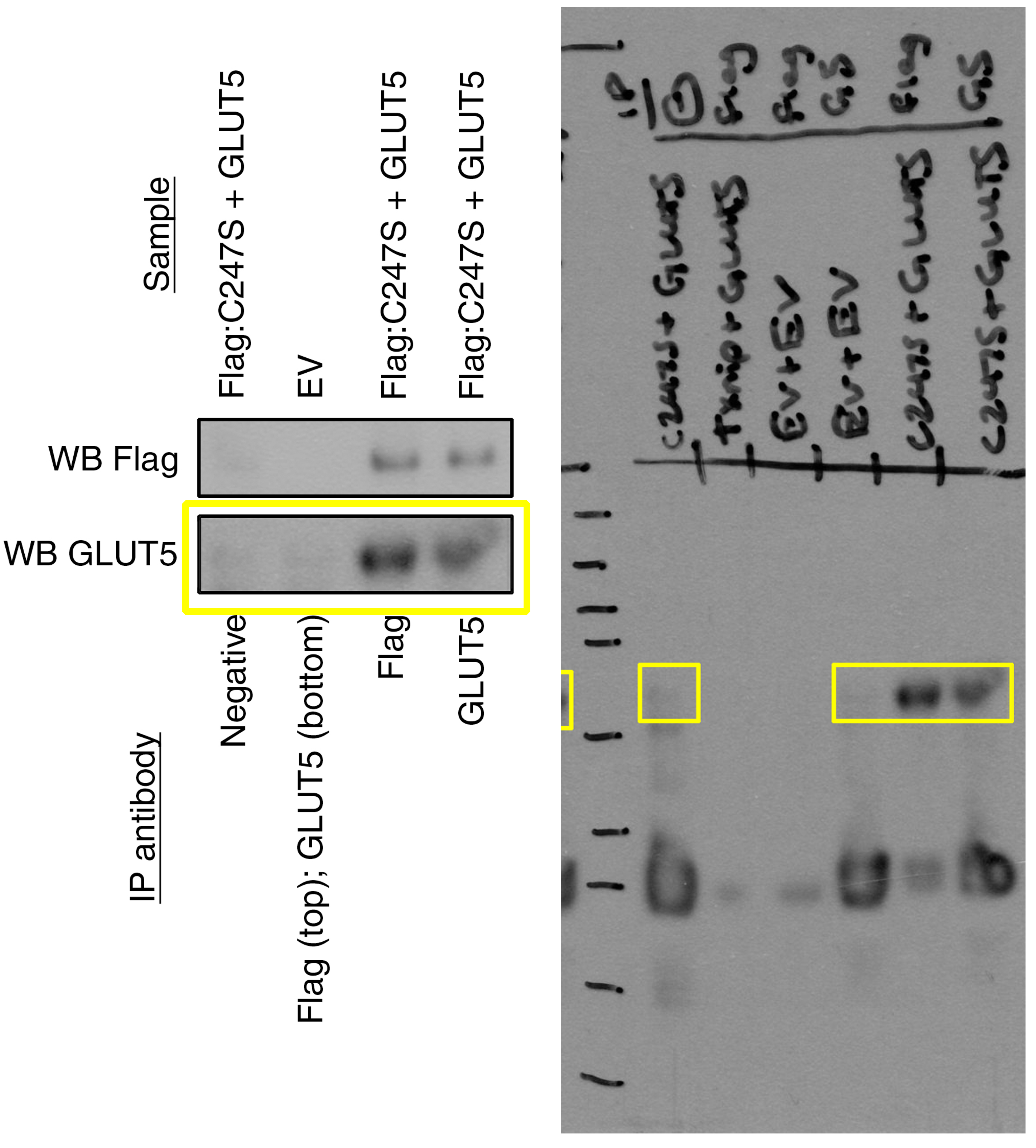


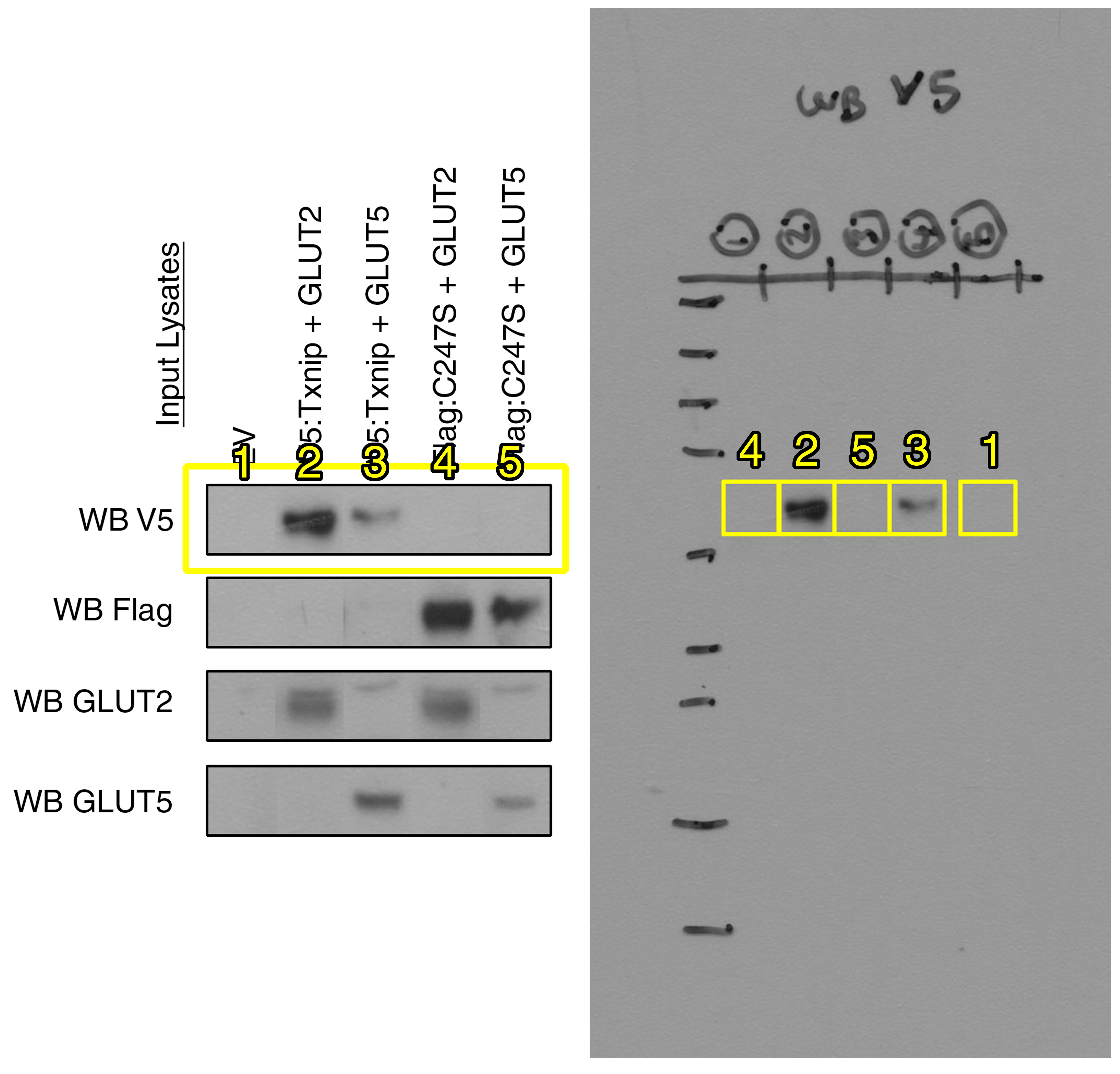

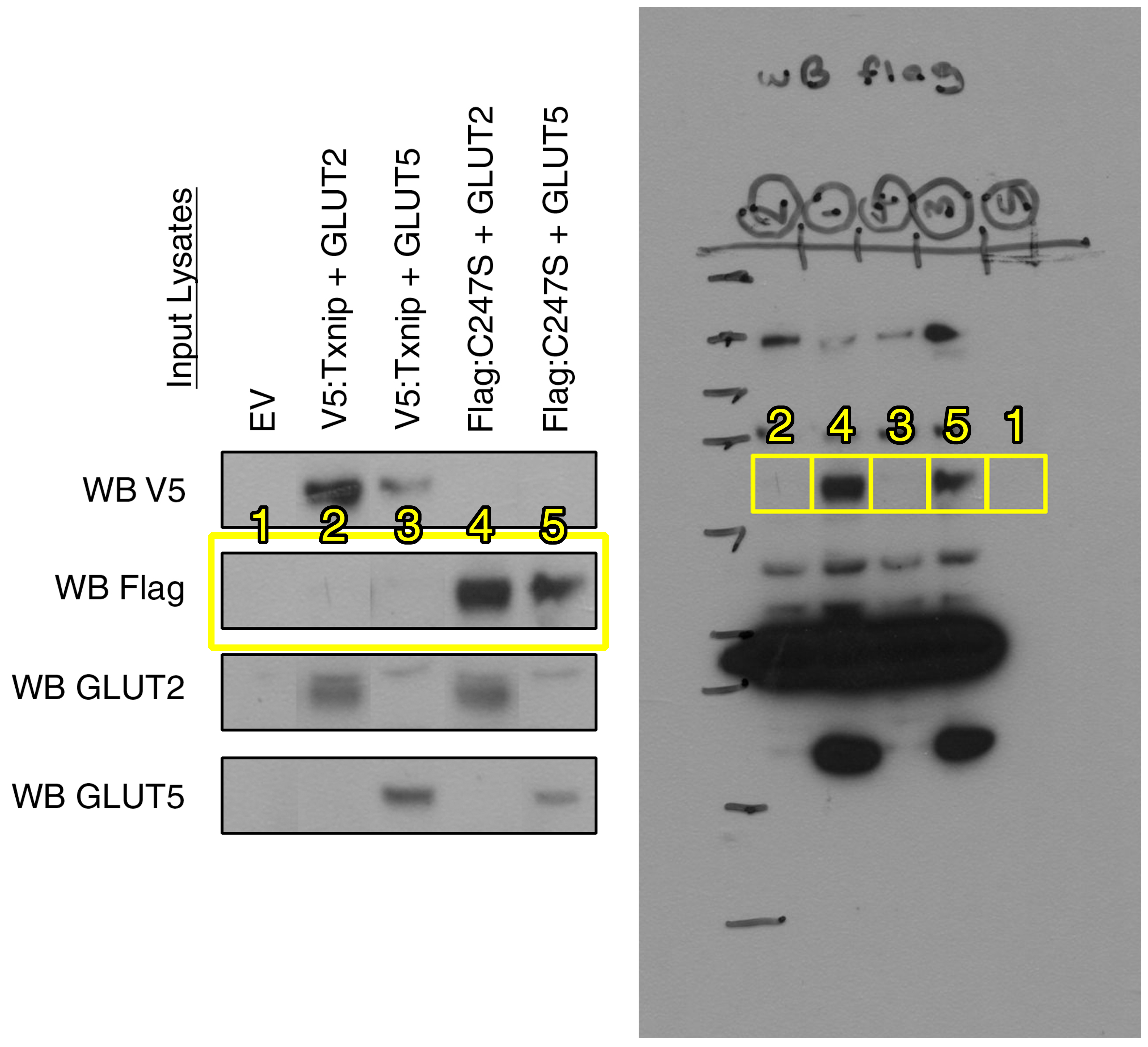


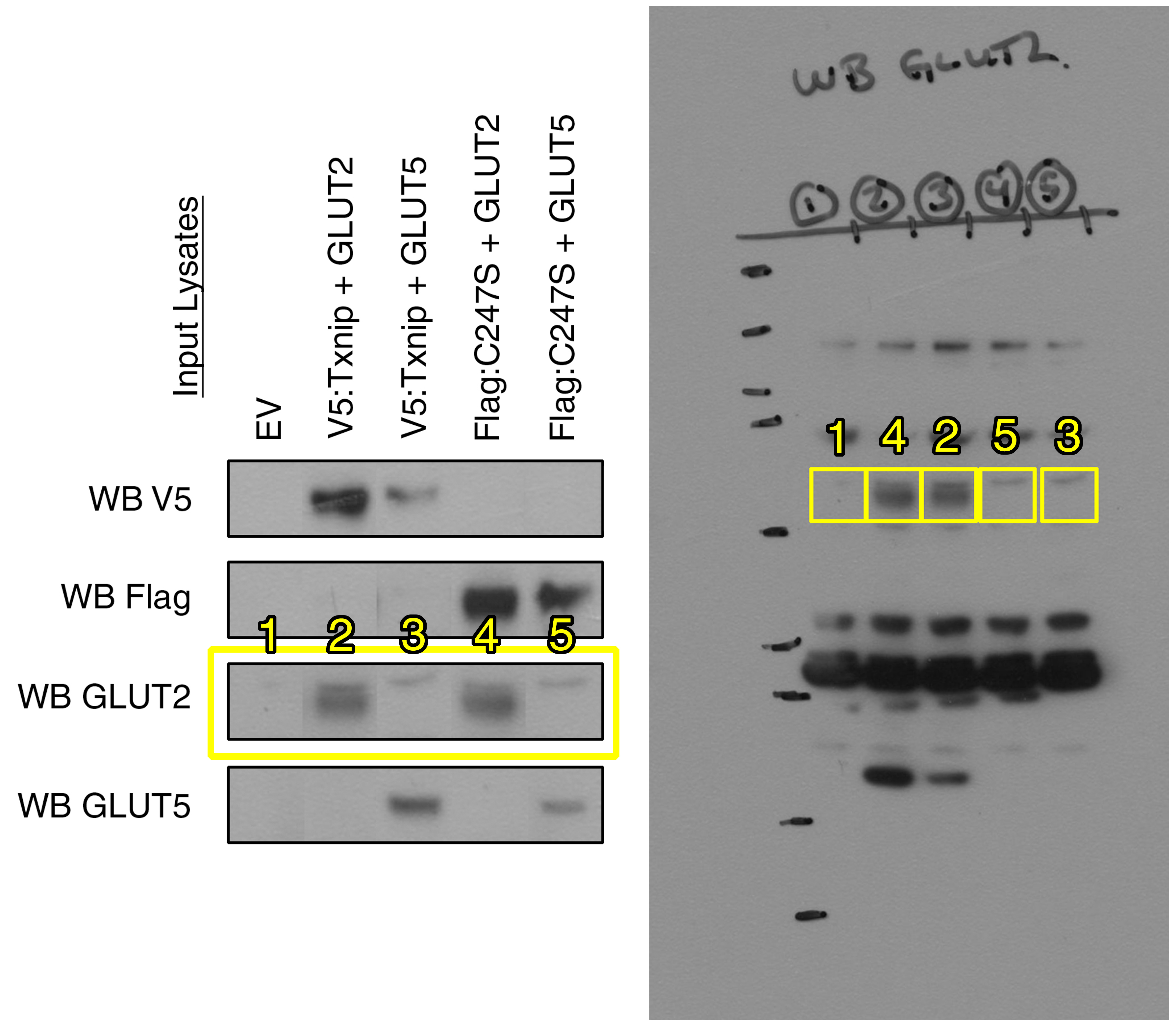

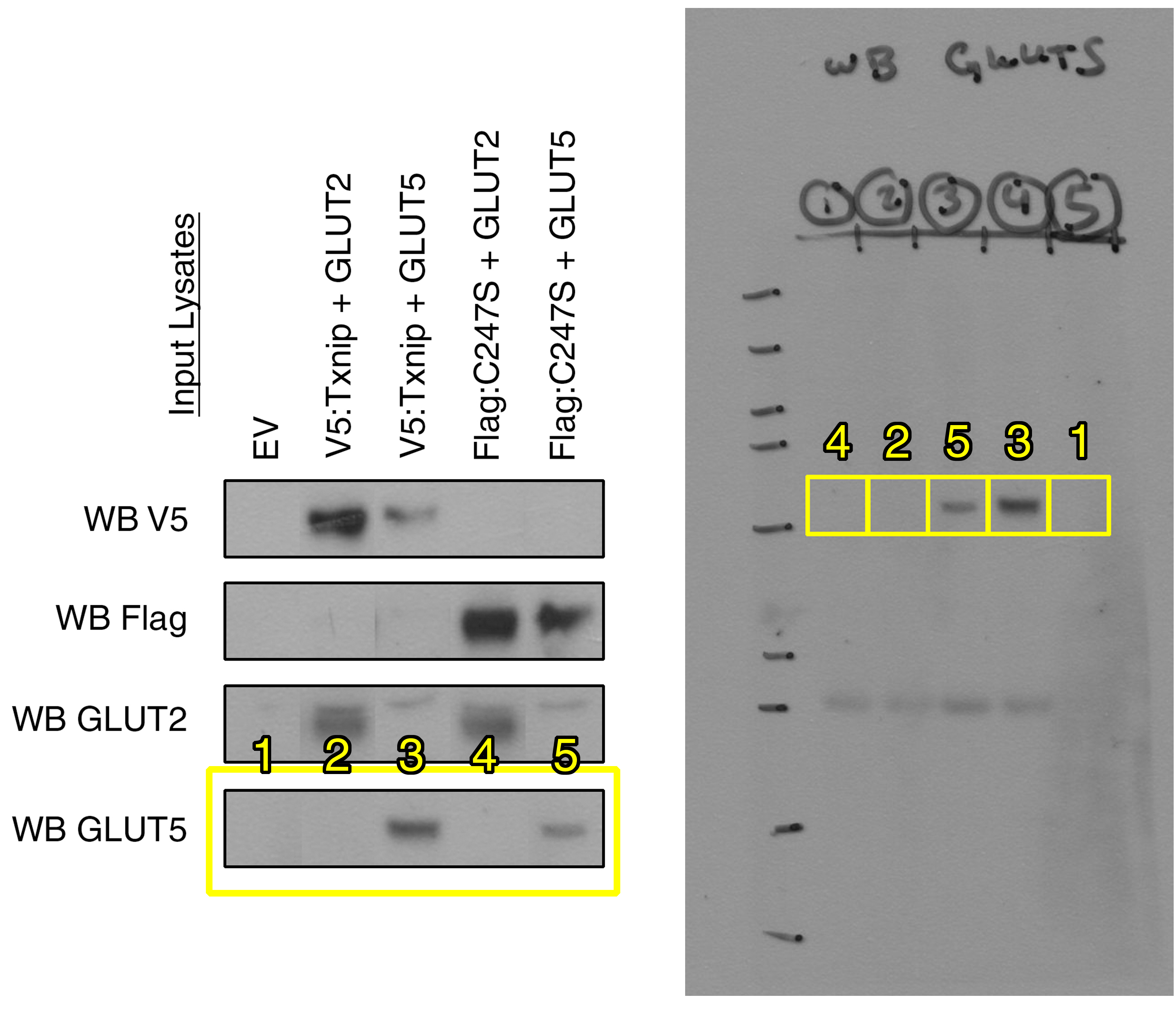

Supplement: Figure 1—source data 1. — The yellow highlighted regions represent the cropped regions. Bands are in the original order of the membrane unless otherwise specified by the numbering above each band. DOI: http://dx.doi.org/10.7554/eLife.18313.004 [file elife-18313-fig1-data1.docx]
