# Supplementary material for: Diabetes regulates fructose absorption through thioredoxin-interacting protein
Source: eLife. 2016 Oct 11;5:e18313. doi: 10.7554/eLife.18313 (PMC5059142; doi:10.7554/eLife.18313)
Supplement: Figure 2—source data 1. — These tables represent the statistical analysis conducted on the raw data collected for Figure 2 using GraphPad Prism 5. DOI: http://dx.doi.org/10.7554/eLife.18313.006 [file elife-18313-fig2-data1.docx]

**Figure 2 source data 1 | Statistical Analysis of Figure 2**

| **Figure 2a** |  |  |  |  |  |
| --- | --- | --- | --- | --- | --- |
| Table Analyzed | Caco-2 Uptake |  |  |  |  |
|  |  |  |  |  |  |
| One-way analysis of variance |  |  |  |  |  |
| P value | < 0.0001 |  |  |  |  |
| P value summary | *** |  |  |  |  |
| Are means signif. different? (P < 0.05) | Yes |  |  |  |  |
| Number of groups | 8 |  |  |  |  |
| F | 24 |  |  |  |  |
| R square | 0.8077 |  |  |  |  |
|  |  |  |  |  |  |
| Bartlett's test for equal variances |  |  |  |  |  |
| Bartlett's statistic (corrected) | 13.24 |  |  |  |  |
| P value | 0.0666 |  |  |  |  |
| P value summary | ns |  |  |  |  |
| Do the variances differ signif. (P < 0.05) | No |  |  |  |  |
|  |  |  |  |  |  |
| ANOVA Table | SS | df | MS |  |  |
| Treatment (between columns) | 50100000 | 7 | 7157000 |  |  |
| Residual (within columns) | 11930000 | 40 | 298197 |  |  |
| Total | 62020000 | 47 |  |  |  |
| **Figure 2a** |  |  |  |  |  |
| Bonferroni's Multiple Comparison Test | Mean Diff. | t | Significant? P < 0.05? | Summary | 95% CI of diff |
| Negative vs +EV | -316 | 1.002 | No | ns | -1371 to 739.3 |
| Negative vs +GLUT2 | -988.7 | 3.136 | No | ns | -2044 to 66.59 |
| Negative vs +GLUT5 | -999.9 | 3.171 | No | ns | -2055 to 55.46 |
| Negative vs +Txnip | -1340 | 4.249 | Yes | ** | -2395 to -284.2 |
| Negative vs +Txnip/+GLUT2 | -1969 | 6.246 | Yes | *** | -3025 to -913.9 |
| Negative vs +Txnip/+GLUT5 | -2260 | 7.167 | Yes | *** | -3315 to -1204 |
| Negative vs +Txnip/+GLUT2/+GLUT5 | -3354 | 10.64 | Yes | *** | -4409 to -2299 |
| +EV vs +GLUT2 | -672.7 | 2.134 | No | ns | -1728 to 382.6 |
| +EV vs +GLUT5 | -683.8 | 2.169 | No | ns | -1739 to 371.5 |
| +EV vs +Txnip | -1023 | 3.246 | No | ns | -2079 to 31.83 |
| +EV vs +Txnip/+GLUT2 | -1653 | 5.244 | Yes | *** | -2709 to -597.8 |
| +EV vs +Txnip/+GLUT5 | -1944 | 6.165 | Yes | *** | -2999 to -888.2 |
| +EV vs +Txnip/+GLUT2/+GLUT5 | -3038 | 9.636 | Yes | *** | -4093 to -1983 |
| +GLUT2 vs +GLUT5 | -11.13 | 0.03529 | No | ns | -1066 to 1044 |
| +GLUT2 vs +Txnip | -350.8 | 1.113 | No | ns | -1406 to 704.5 |
| +GLUT2 vs +Txnip/+GLUT2 | -980.5 | 3.11 | No | ns | -2036 to 74.86 |
| +GLUT2 vs +Txnip/+GLUT5 | -1271 | 4.031 | Yes | ** | -2326 to -215.5 |
| +GLUT2 vs +Txnip/+GLUT2/+GLUT5 | -2365 | 7.503 | Yes | *** | -3421 to -1310 |
| +GLUT5 vs +Txnip | -339.7 | 1.077 | No | ns | -1395 to 715.7 |
| +GLUT5 vs +Txnip/+GLUT2 | -969.3 | 3.075 | No | ns | -2025 to 85.99 |
| +GLUT5 vs +Txnip/+GLUT5 | -1260 | 3.996 | Yes | ** | -2315 to -204.4 |
| +GLUT5 vs +Txnip/+GLUT2/+GLUT5 | -2354 | 7.467 | Yes | *** | -3410 to -1299 |
| +Txnip vs +Txnip/+GLUT2 | -629.7 | 1.997 | No | ns | -1685 to 425.7 |
| +Txnip vs +Txnip/+GLUT5 | -920 | 2.918 | No | ns | -1975 to 135.3 |
| +Txnip vs +Txnip/+GLUT2/+GLUT5 | -2015 | 6.39 | Yes | *** | -3070 to -959.3 |
| +Txnip/+GLUT2 vs +Txnip/+GLUT5 | -290.4 | 0.921 | No | ns | -1346 to 765.0 |
| +Txnip/+GLUT2 vs +Txnip/+GLUT2/+GLUT5 | -1385 | 4.393 | Yes | ** | -2440 to -329.6 |
| +Txnip/+GLUT5 vs +Txnip/+GLUT2/+GLUT5 | -1095 | 3.472 | Yes | * | -2150 to -39.25 |
|  |  |  |  |  |  |
| Table Analyzed | Ratio fold change |  |  |  |  |
| Column A | +Txnip/+GLUT2 |  |  |  |  |
| vs | vs |  |  |  |  |
| Column B | +Txnip/+GLUT5 |  |  |  |  |
|  |  |  |  |  |  |
| Unpaired t test |  |  |  |  |  |
| P value | 0.1937 |  |  |  |  |
| P value summary | ns |  |  |  |  |
| Are means signif. different? (P < 0.05) | No |  |  |  |  |
| One- or two-tailed P value? | Two-tailed |  |  |  |  |
| t, df | t=1.393 df=10 |  |  |  |  |
|  |  |  |  |  |  |
| How big is the difference? |  |  |  |  |  |
| Mean ± SEM of column A | 0.6650 ± 0.02980 N=6 |  |  |  |  |
| Mean ± SEM of column B | 0.7353 ± 0.04065 N=6 |  |  |  |  |
| Difference between means | -0.07023 ± 0.05040 |  |  |  |  |
| 95% confidence interval | -0.1825 to 0.04207 |  |  |  |  |
| R square | 0.1626 |  |  |  |  |
|  |  |  |  |  |  |
| F test to compare variances |  |  |  |  |  |
| F,DFn, Dfd | 1.860, 5, 5 |  |  |  |  |
| P value | 0.5122 |  |  |  |  |
| P value summary | ns |  |  |  |  |
| Are variances significantly different? | No |  |  |  |  |

| Bonferroni's Multiple Comparison Test | Mean Diff. | t | Significant? P < 0.05? | Summary | 95% CI of diff |
| --- | --- | --- | --- | --- | --- |
| WT vs KO | 94.02 | 9.576 | Yes | *** | 67.57 to 120.5 |
| WT vs KO + Txnip | 22.86 | 2.329 | No | ns | -3.584 to 49.31 |
| KO vs KO + Txnip | -71.16 | 7.248 | Yes | *** | -97.60 to -44.71 |

| **Figure 2b** |  |  |  |  |  |
| --- | --- | --- | --- | --- | --- |
| Table Analyzed | MEF Fructose Uptake |  |  |  |  |
|  |  |  |  |  |  |
| One-way analysis of variance |  |  |  |  |  |
| P value | < 0.0001 |  |  |  |  |
| P value summary | *** |  |  |  |  |
| Are means signif. different? (P < 0.05) | Yes |  |  |  |  |
| Number of groups | 3 |  |  |  |  |
| F | 26.95 |  |  |  |  |
| R square | 0.7823 |  |  |  |  |
|  |  |  |  |  |  |
| Bartlett's test for equal variances |  |  |  |  |  |
| Bartlett's statistic (corrected) | 1.638 |  |  |  |  |
| P value | 0.4408 |  |  |  |  |
| P value summary | ns |  |  |  |  |
| Do the variances differ signif. (P < 0.05) | No |  |  |  |  |
|  |  |  |  |  |  |
| ANOVA Table | SS | df | MS |  |  |
| Treatment (between columns) | 15930 | 2 | 7965 |  |  |
| Residual (within columns) | 4433 | 15 | 295.5 |  |  |
| Total | 20363 | 17 |  |  |  |
|  |  |  |  |  |  |
| Bonferroni's Multiple Comparison Test | Mean Diff. | t | Significant? P < 0.05? | Summary | 95% CI of diff |
| WT vs Txnip-KO | 67.1 | 6.76 | Yes | *** | 40.36 to 93.84 |
| WT vs Txnip-KO + Txnip | 8.94 | 0.9007 | No | ns | -17.80 to 35.68 |
| Txnip-KO vs Txnip-KO + Txnip | -58.16 | 5.86 | Yes | *** | -84.90 to -31.42 |

| **Figure 2c** | |
| --- | --- |
| Table Analyzed | Ex Vivo Intestinal Fructose Uptake |
| Column A | WT |
| vs | vs |
| Column B | KO |
|  |  |
| Unpaired t test |  |
| P value | < 0.0001 |
| P value summary | *** |
| Are means signif. different? (P < 0.05) | Yes |
| One- or two-tailed P value? | Two-tailed |
| t, df | t=6.518 df=10 |
|  |  |
| How big is the difference? |  |
| Mean ± SEM of column A | 21.07 ± 1.227 N=6 |
| Mean ± SEM of column B | 10.72 ± 1.008 N=6 |
| Difference between means | 10.35 ± 1.588 |
| 95% confidence interval | 6.812 to 13.89 |
| R square | 0.8095 |

| **Figure 2d** |  |
| --- | --- |
| Table Analyzed | Blood |
| Column A | WT |
| vs | vs |
| Column B | Txnip-KO |
|  |  |
| Unpaired t test |  |
| P value | 0.0285 |
| P value summary | * |
| Are means signif. different? (P < 0.05) | Yes |
| One- or two-tailed P value? | Two-tailed |
| t, df | t=2.869 df=6 |
|  |  |
| How big is the difference? |  |
| Mean ± SEM of column A | 1982 ± 263.8 N=4 |
| Mean ± SEM of column B | 1184 ± 88.96 N=4 |
| Difference between means | 798.6 ± 278.4 |
| 95% confidence interval | 117.4 to 1480 |
| R square | 0.5783 |
|  |  |
| Table Analyzed | Liver |
| Column A | WT |
| vs | vs |
| Column B | Txnip-KO |
|  |  |
| Unpaired t test |  |
| P value | 0.0083 |
| P value summary | ** |
| Are means signif. different? (P < 0.05) | Yes |
| One- or two-tailed P value? | Two-tailed |
| t, df | t=3.869 df=6 |
|  |  |
| How big is the difference? |  |
| Mean ± SEM of column A | 2404 ± 296.3 N=4 |
| Mean ± SEM of column B | 1020 ± 200.3 N=4 |
| Difference between means | 1384 ± 357.6 |
| 95% confidence interval | 508.5 to 2259 |
| R square | 0.7139 |
|  |  |
| Table Analyzed | Heart |
| Column A | WT |
| vs | vs |
| Column B | Txnip-KO |
|  |  |
| Unpaired t test |  |
| P value | 0.0104 |
| P value summary | * |
| Are means signif. different? (P < 0.05) | Yes |
| One- or two-tailed P value? | Two-tailed |
| t, df | t=3.144 df=10 |
|  |  |
| How big is the difference? |  |
| Mean ± SEM of column A | 2103 ± 384.8 N=4 |
| Mean ± SEM of column B | 769.9 ± 178.1 N=4 |
| Difference between means | 1333 ± 424.1 |
| 95% confidence interval | 388.4 to 2278 |
| R square | 0.4971 |
|  |  |
| Table Analyzed | Kidney |
| Column A | WT |
| vs | vs |
| Column B | Txnip-KO |
|  |  |
| Unpaired t test |  |
| P value | 0.0448 |
| P value summary | * |
| Are means signif. different? (P < 0.05) | Yes |
| One- or two-tailed P value? | Two-tailed |
| t, df | t=2.529 df=6 |
|  |  |
| How big is the difference? |  |
| Mean ± SEM of column A | 1223 ± 113.0 N=4 |
| Mean ± SEM of column B | 913.0 ± 47.79 N=4 |
| Difference between means | 310.3 ± 122.7 |
| 95% confidence interval | 10.04 to 610.6 |
| R square | 0.5159 |
|  |  |
| Table Analyzed | Rectus Femoris |
| Column A | WT |
| vs | vs |
| Column B | Txnip-KO |
|  |  |
| Unpaired t test |  |
| P value | 0.6198 |
| P value summary | ns |
| Are means signif. different? (P < 0.05) | No |
| One- or two-tailed P value? | Two-tailed |
| t, df | t=0.5229 df=6 |
|  |  |
| How big is the difference? |  |
| Mean ± SEM of column A | 305.8 ± 42.05 N=4 |
| Mean ± SEM of column B | 357.2 ± 88.96 N=4 |
| Difference between means | -51.45 ± 98.39 |
| 95% confidence interval | -292.2 to 189.3 |
| R square | 0.04358 |
|  |  |
| Table Analyzed | Brain |
| Column A | WT |
| vs | vs |
| Column B | Txnip-KO |
|  |  |
| Unpaired t test |  |
| P value | 0.0904 |
| P value summary | ns |
| Are means signif. different? (P < 0.05) | No |
| One- or two-tailed P value? | Two-tailed |
| t, df | t=1.874 df=10 |
|  |  |
| How big is the difference? |  |
| Mean ± SEM of column A | 1210 ± 165.7 N=4 |
| Mean ± SEM of column B | 837.1 ± 110.5 N=6 |
| Difference between means | 373.2 ± 199.2 |
| 95% confidence interval | -70.52 to 816.9 |
| R square | 0.2599 |

**Figure 2 source data 1 | Statistical Analysis of Figure 2.** These tables represent the statistical analysis conducted on the raw data collected for Figure 2 using GraphPad Prism 5.
