# Supplementary material for: Diabetes regulates fructose absorption through thioredoxin-interacting protein
Source: eLife. 2016 Oct 11;5:e18313. doi: 10.7554/eLife.18313 (PMC5059142; doi:10.7554/eLife.18313)
Supplement: Figure 2—source data 2. — These tables represent the statistical analysis conducted on the raw data collected for Figure 2—figure supplement 1 using GraphPad Prism 5. DOI: http://dx.doi.org/10.7554/eLife.18313.007 [file elife-18313-fig2-data2.docx]

**Figure 2-source data 2 | Statistical Analysis for Figure 2-figure supplement 1**

| Table Analyzed | Peripheral Blood |
| --- | --- |
| Column A | WT |
| vs | vs |
| Column B | KO |
|  |  |
| Unpaired t test |  |
| P value | 0.7616 |
| P value summary | ns |
| Are means signif. different? (P < 0.05) | No |
| One- or two-tailed P value? | Two-tailed |
| t, df | t=0.3093 df=14 |
|  |  |
| How big is the difference? |  |
| Mean ± SEM of column A | 0.9828 ± 0.08434 N=8 |
| Mean ± SEM of column B | 1.017 ± 0.07297 N=8 |
| Difference between means | -0.03450 ± 0.1115 |
| 95% confidence interval | -0.2737 to 0.2047 |
| R square | 0.006789 |
|  |  |
|  |  |
| Table Analyzed | Liver |
| Column A | WT |
| vs | vs |
| Column B | KO |
|  |  |
| Unpaired t test |  |
| P value | 0.7463 |
| P value summary | ns |
| Are means signif. different? (P < 0.05) | No |
| One- or two-tailed P value? | Two-tailed |
| t, df | t=0.3300 df=14 |
|  |  |
| How big is the difference? |  |
| Mean ± SEM of column A | 1.011 ± 0.1241 N=8 |
| Mean ± SEM of column B | 1.070 ± 0.1304 N=8 |
| Difference between means | -0.05941 ± 0.1800 |
| 95% confidence interval | -0.4455 to 0.3267 |
| R square | 0.007718 |
|  |  |
|  |  |
| Table Analyzed | Heart |
| Column A | WT |
| vs | vs |
| Column B | KO |
|  |  |
| Unpaired t test |  |
| P value | 0.7322 |
| P value summary | ns |
| Are means signif. different? (P < 0.05) | No |
| One- or two-tailed P value? | Two-tailed |
| t, df | t=0.3491 df=14 |
|  |  |
| How big is the difference? |  |
| Mean ± SEM of column A | 0.5433 ± 0.07780 N=8 |
| Mean ± SEM of column B | 0.5912 ± 0.1132 N=8 |
| Difference between means | -0.04795 ± 0.1374 |
| 95% confidence interval | -0.3426 to 0.2467 |
| R square | 0.008629 |
|  |  |
|  |  |
| Table Analyzed | Kidney |
| Column A | WT |
| vs | vs |
| Column B | KO |
|  |  |
| Unpaired t test |  |
| P value | 0.2518 |
| P value summary | ns |
| Are means signif. different? (P < 0.05) | No |
| One- or two-tailed P value? | Two-tailed |
| t, df | t=1.195 df=14 |
|  |  |
| How big is the difference? |  |
| Mean ± SEM of column A | 1.507 ± 0.1727 N=8 |
| Mean ± SEM of column B | 1.249 ± 0.1294 N=8 |
| Difference between means | 0.2579 ± 0.2158 |
| 95% confidence interval | -0.2049 to 0.7207 |
| R square | 0.0926 |
|  |  |
|  |  |
| Table Analyzed | Brain |
| Column A | WT |
| vs | vs |
| Column B | KO |
|  |  |
| Unpaired t test |  |
| P value | 0.6276 |
| P value summary | ns |
| Are means signif. different? (P < 0.05) | No |
| One- or two-tailed P value? | Two-tailed |
| t, df | t=0.4960 df=14 |
|  |  |
| How big is the difference? |  |
| Mean ± SEM of column A | 0.6900 ± 0.07580 N=8 |
| Mean ± SEM of column B | 0.6445 ± 0.05184 N=8 |
| Difference between means | 0.04555 ± 0.09183 |
| 95% confidence interval | -0.1514 to 0.2425 |
| R square | 0.01727 |
|  |  |
|  |  |
| Table Analyzed | Rectus Femoris |
| Column A | WT |
| vs | vs |
| Column B | KO |
|  |  |
| Unpaired t test |  |
| P value | 0.5067 |
| P value summary | ns |
| Are means signif. different? (P < 0.05) | No |
| One- or two-tailed P value? | Two-tailed |
| t, df | t=0.6815 df=14 |
|  |  |
| How big is the difference? |  |
| Mean ± SEM of column A | 0.2999 ± 0.02808 N=8 |
| Mean ± SEM of column B | 0.3318 ± 0.03743 N=8 |
| Difference between means | -0.03189 ± 0.04680 |
| 95% confidence interval | -0.1323 to 0.06849 |
| R square | 0.03211 |
