# Supplementary material for: Diabetes regulates fructose absorption through thioredoxin-interacting protein
Source: eLife. 2016 Oct 11;5:e18313. doi: 10.7554/eLife.18313 (PMC5059142; doi:10.7554/eLife.18313)
Supplement: Figure 3—source data 1. — These tables represent the statistical analysis conducted on the raw data collected for Figure 3 using GraphPad Prism 5. DOI: http://dx.doi.org/10.7554/eLife.18313.011 [file elife-18313-fig3-data1.docx]

**Figure 3-source data 1 | Statistical Analysis for Figure 3**

| **Figure 3a** | | | | | |
| --- | --- | --- | --- | --- | --- |
| Bonferroni's Multiple Comparison Test | Mean Diff. | t | Significant? P < 0.05? | Summary | 95% CI of diff |
| WT, RD vs WT, FSD | -7.734 | 3.412 | Yes | * | -14.41 to -1.060 |
| WT, RD vs Txnip-KO, RD | 1.486 | 0.6553 | No | ns | -5.188 to 8.160 |
| WT, RD vs Txnip-KO, FSD | 0.03572 | 0.01658 | No | ns | -6.306 to 6.377 |
| WT, FSD vs Txnip-KO, RD | 9.22 | 3.765 | Yes | ** | 2.011 to 16.43 |
| WT, FSD vs Txnip-KO, FSD | 7.77 | 3.314 | Yes | * | 0.8680 to 14.67 |
| Txnip-KO, RD vs Txnip-KO, FSD | -1.45 | 0.6185 | No | ns | -8.352 to 5.452 |
|  |  |  |  |  |  |
| WT vs KO |  |  |  |  |  |
| Row Factor | WT | KO | Difference | 95% CI of diff. |  |
| RD | 31.59 | 30.1 | -1.486 | -7.003 to 4.031 |  |
| FSD | 39.32 | 31.55 | -7.77 | -13.48 to -2.065 |  |
|  |  |  |  |  |  |
| Row Factor | Difference | t | P value | Summary |  |
| RD | -1.486 | 0.6553 | P > 0.05 | ns |  |
| FSD | -7.77 | 3.314 | P<0.01 | ** |  |

| **Figure 3b** |  |  |  |  |
| --- | --- | --- | --- | --- |
| Bonferroni posttests |  |  |  |  |
|  |  |  |  |  |
| Wild Type, Normal Diet vs Wild Type, Fructose Diet |  |  |  |  |
| Column Factor | Wild Type, Normal Diet | Wild Type, Fructose Diet | Difference | 95% CI of diff. |
| 0.0 | 124 | 138 | 14 | -39.08 to 67.08 |
| 15.00 | 98.14 | 128.3 | 30.14 | -22.93 to 83.22 |
| 30.00 | 74 | 97.71 | 23.71 | -29.36 to 76.79 |
| 60.00 | 51.14 | 98.86 | 47.71 | -5.361 to 100.8 |
| 120.0 | 64.57 | 125.9 | 61.29 | 8.210 to 114.4 |
|  |  |  |  |  |
| Column Factor | Difference | t | P value | Summary |
| 0.0 | 14 | 0.8551 | P > 0.05 | ns |
| 15.00 | 30.14 | 1.841 | P > 0.05 | ns |
| 30.00 | 23.71 | 1.448 | P > 0.05 | ns |
| 60.00 | 47.71 | 2.914 | P < 0.05 | * |
| 120.0 | 61.29 | 3.743 | P<0.01 | ** |
|  |  |  |  |  |
| Wild Type, Normal Diet vs Txnip-KO, Normal Diet |  |  |  |  |
| Column Factor | Wild Type, Normal Diet | Txnip-KO, Normal Diet | Difference | 95% CI of diff. |
| 0.0 | 124 | 63.75 | -60.25 | -122.5 to 1.986 |
| 15.00 | 98.14 | 64 | -34.14 | -96.38 to 28.09 |
| 30.00 | 74 | 35.75 | -38.25 | -100.5 to 23.99 |
| 60.00 | 51.14 | 26.5 | -24.64 | -86.88 to 37.59 |
| 120.0 | 64.57 | 26.25 | -38.32 | -100.6 to 23.91 |
|  |  |  |  |  |
| Column Factor | Difference | t | P value | Summary |
| 0.0 | -60.25 | 3.138 | P < 0.05 | * |
| 15.00 | -34.14 | 1.778 | P > 0.05 | ns |
| 30.00 | -38.25 | 1.992 | P > 0.05 | ns |
| 60.00 | -24.64 | 1.284 | P > 0.05 | ns |
| 120.0 | -38.32 | 1.996 | P > 0.05 | ns |
|  |  |  |  |  |
| Wild Type, Normal Diet vs Txnip-KO, Fructose Diet |  |  |  |  |
| Column Factor | Wild Type, Normal Diet | Txnip-KO, Fructose Diet | Difference | 95% CI of diff. |
| 0.0 | 124 | 70.5 | -53.5 | -115.7 to 8.736 |
| 15.00 | 98.14 | 71 | -27.14 | -89.38 to 35.09 |
| 30.00 | 74 | 49.5 | -24.5 | -86.74 to 37.74 |
| 60.00 | 51.14 | 29.75 | -21.39 | -83.63 to 40.84 |
| 120.0 | 64.57 | 35.5 | -29.07 | -91.31 to 33.16 |
|  |  |  |  |  |
| Column Factor | Difference | t | P value | Summary |
| 0.0 | -53.5 | 2.787 | P < 0.05 | * |
| 15.00 | -27.14 | 1.414 | P > 0.05 | ns |
| 30.00 | -24.5 | 1.276 | P > 0.05 | ns |
| 60.00 | -21.39 | 1.114 | P > 0.05 | ns |
| 120.0 | -29.07 | 1.514 | P > 0.05 | ns |
|  |  |  |  |  |
| Wild Type, Fructose Diet vs Txnip-KO, Normal Diet |  |  |  |  |
| Column Factor | Wild Type, Fructose Diet | Txnip-KO, Normal Diet | Difference | 95% CI of diff. |
| 0.0 | 138 | 63.75 | -74.25 | -136.5 to -12.01 |
| 15.00 | 128.3 | 64 | -64.29 | -126.5 to -2.049 |
| 30.00 | 97.71 | 35.75 | -61.96 | -124.2 to 0.2721 |
| 60.00 | 98.86 | 26.5 | -72.36 | -134.6 to -10.12 |
| 120.0 | 125.9 | 26.25 | -99.61 | -161.8 to -37.37 |
|  |  |  |  |  |
| Column Factor | Difference | t | P value | Summary |
| 0.0 | -74.25 | 3.867 | P<0.01 | ** |
| 15.00 | -64.29 | 3.348 | P<0.01 | ** |
| 30.00 | -61.96 | 3.228 | P<0.01 | ** |
| 60.00 | -72.36 | 3.769 | P<0.01 | ** |
| 120.0 | -99.61 | 5.188 | P<0.001 | *** |
|  |  |  |  |  |
| Wild Type, Fructose Diet vs Txnip-KO, Fructose Diet |  |  |  |  |
| Column Factor | Wild Type, Fructose Diet | Txnip-KO, Fructose Diet | Difference | 95% CI of diff. |
| 0.0 | 138 | 70.5 | -67.5 | -129.7 to -5.264 |
| 15.00 | 128.3 | 71 | -57.29 | -119.5 to 4.951 |
| 30.00 | 97.71 | 49.5 | -48.21 | -110.5 to 14.02 |
| 60.00 | 98.86 | 29.75 | -69.11 | -131.3 to -6.871 |
| 120.0 | 125.9 | 35.5 | -90.36 | -152.6 to -28.12 |
|  |  |  |  |  |
| Column Factor | Difference | t | P value | Summary |
| 0.0 | -67.5 | 3.516 | P<0.01 | ** |
| 15.00 | -57.29 | 2.984 | P < 0.05 | * |
| 30.00 | -48.21 | 2.511 | P > 0.05 | ns |
| 60.00 | -69.11 | 3.6 | P<0.01 | ** |
| 120.0 | -90.36 | 4.706 | P<0.001 | *** |
|  |  |  |  |  |
| Txnip-KO, Normal Diet vs Txnip-KO, Fructose Diet |  |  |  |  |
| Column Factor | Txnip-KO, Normal Diet | Txnip-KO, Fructose Diet | Difference | 95% CI of diff. |
| 0.0 | 63.75 | 70.5 | 6.75 | -63.46 to 76.96 |
| 15.00 | 64 | 71 | 7 | -63.21 to 77.21 |
| 30.00 | 35.75 | 49.5 | 13.75 | -56.46 to 83.96 |
| 60.00 | 26.5 | 29.75 | 3.25 | -66.96 to 73.46 |
| 120.0 | 26.25 | 35.5 | 9.25 | -60.96 to 79.46 |
|  |  |  |  |  |
| Column Factor | Difference | t | P value | Summary |
| 0.0 | 6.75 | 0.3117 | P > 0.05 | ns |
| 15.00 | 7 | 0.3232 | P > 0.05 | ns |
| 30.00 | 13.75 | 0.6348 | P > 0.05 | ns |
| 60.00 | 3.25 | 0.1501 | P > 0.05 | ns |
| 120.0 | 9.25 | 0.4271 | P > 0.05 | ns |

| **Figure 3c** |  |  |  |  |
| --- | --- | --- | --- | --- |
| Bonferroni posttests |  |  |  |  |
|  |  |  |  |  |
| WT, RD vs WT, FSD |  |  |  |  |
| Column Factor | WT, RD | WT, FSD | Difference | 95% CI of diff. |
| 0.0 | 132.3 | 143.6 | 11.29 | -58.62 to 81.20 |
| 15.00 | 248.9 | 265.7 | 16.86 | -53.05 to 86.77 |
| 30.00 | 224.4 | 287.7 | 63.29 | -6.624 to 133.2 |
| 60.00 | 185 | 292.9 | 107.9 | 37.95 to 177.8 |
| 120.0 | 141.4 | 210.7 | 69.29 | -0.6237 to 139.2 |
|  |  |  |  |  |
| Column Factor | Difference | t | P value | Summary |
| 0.0 | 11.29 | 0.5233 | P > 0.05 | ns |
| 15.00 | 16.86 | 0.7817 | P > 0.05 | ns |
| 30.00 | 63.29 | 2.935 | P < 0.05 | * |
| 60.00 | 107.9 | 5.001 | P<0.001 | *** |
| 120.0 | 69.29 | 3.213 | P<0.01 | ** |
|  |  |  |  |  |
| WT, RD vs Txnip-KO, RD |  |  |  |  |
| Column Factor | WT, RD | Txnip-KO, RD | Difference | 95% CI of diff. |
| 0.0 | 132.3 | 60 | -72.29 | -154.3 to 9.690 |
| 15.00 | 248.9 | 165 | -83.86 | -165.8 to -1.881 |
| 30.00 | 224.4 | 152.8 | -71.68 | -153.7 to 10.30 |
| 60.00 | 185 | 104.8 | -80.25 | -162.2 to 1.726 |
| 120.0 | 141.4 | 54.75 | -86.68 | -168.7 to -4.703 |
|  |  |  |  |  |
| Column Factor | Difference | t | P value | Summary |
| 0.0 | -72.29 | 2.859 | P < 0.05 | * |
| 15.00 | -83.86 | 3.316 | P<0.01 | ** |
| 30.00 | -71.68 | 2.835 | P < 0.05 | * |
| 60.00 | -80.25 | 3.173 | P < 0.05 | * |
| 120.0 | -86.68 | 3.428 | P<0.01 | ** |
|  |  |  |  |  |
| WT, RD vs Txnip-KO, FSD |  |  |  |  |
| Column Factor | WT, RD | Txnip-KO, FSD | Difference | 95% CI of diff. |
| 0.0 | 132.3 | 63.25 | -69.04 | -151.0 to 12.94 |
| 15.00 | 248.9 | 179.8 | -69.11 | -151.1 to 12.87 |
| 30.00 | 224.4 | 153.5 | -70.93 | -152.9 to 11.05 |
| 60.00 | 185 | 135.3 | -49.75 | -131.7 to 32.23 |
| 120.0 | 141.4 | 61.75 | -79.68 | -161.7 to 2.297 |
|  |  |  |  |  |
| Column Factor | Difference | t | P value | Summary |
| 0.0 | -69.04 | 2.73 | P < 0.05 | * |
| 15.00 | -69.11 | 2.733 | P < 0.05 | * |
| 30.00 | -70.93 | 2.805 | P < 0.05 | * |
| 60.00 | -49.75 | 1.967 | P > 0.05 | ns |
| 120.0 | -79.68 | 3.151 | P < 0.05 | * |
|  |  |  |  |  |
| WT, FSD vs Txnip-KO, RD |  |  |  |  |
| Column Factor | WT, FSD | Txnip-KO, RD | Difference | 95% CI of diff. |
| 0.0 | 143.6 | 60 | -83.57 | -165.5 to -1.595 |
| 15.00 | 265.7 | 165 | -100.7 | -182.7 to -18.74 |
| 30.00 | 287.7 | 152.8 | -135 | -216.9 to -52.99 |
| 60.00 | 292.9 | 104.8 | -188.1 | -270.1 to -106.1 |
| 120.0 | 210.7 | 54.75 | -156 | -237.9 to -73.99 |
|  |  |  |  |  |
| Column Factor | Difference | t | P value | Summary |
| 0.0 | -83.57 | 3.305 | P<0.01 | ** |
| 15.00 | -100.7 | 3.983 | P<0.001 | *** |
| 30.00 | -135 | 5.337 | P<0.001 | *** |
| 60.00 | -188.1 | 7.439 | P<0.001 | *** |
| 120.0 | -156 | 6.168 | P<0.001 | *** |
|  |  |  |  |  |
| WT, FSD vs Txnip-KO, FSD |  |  |  |  |
| Column Factor | WT, FSD | Txnip-KO, FSD | Difference | 95% CI of diff. |
| 0.0 | 143.6 | 63.25 | -80.32 | -162.3 to 1.655 |
| 15.00 | 265.7 | 179.8 | -85.96 | -167.9 to -3.988 |
| 30.00 | 287.7 | 153.5 | -134.2 | -216.2 to -52.24 |
| 60.00 | 292.9 | 135.3 | -157.6 | -239.6 to -75.63 |
| 120.0 | 210.7 | 61.75 | -149 | -230.9 to -66.99 |
|  |  |  |  |  |
| Column Factor | Difference | t | P value | Summary |
| 0.0 | -80.32 | 3.176 | P < 0.05 | * |
| 15.00 | -85.96 | 3.399 | P<0.01 | ** |
| 30.00 | -134.2 | 5.307 | P<0.001 | *** |
| 60.00 | -157.6 | 6.233 | P<0.001 | *** |
| 120.0 | -149 | 5.891 | P<0.001 | *** |
|  |  |  |  |  |
| Txnip-KO, RD vs Txnip-KO, FSD |  |  |  |  |
| Column Factor | Txnip-KO, RD | Txnip-KO, FSD | Difference | 95% CI of diff. |
| 0.0 | 60 | 63.25 | 3.25 | -89.23 to 95.73 |
| 15.00 | 165 | 179.8 | 14.75 | -77.73 to 107.2 |
| 30.00 | 152.8 | 153.5 | 0.75 | -91.73 to 93.23 |
| 60.00 | 104.8 | 135.3 | 30.5 | -61.98 to 123.0 |
| 120.0 | 54.75 | 61.75 | 7 | -85.48 to 99.48 |
|  |  |  |  |  |
| Column Factor | Difference | t | P value | Summary |
| 0.0 | 3.25 | 0.1139 | P > 0.05 | ns |
| 15.00 | 14.75 | 0.517 | P > 0.05 | ns |
| 30.00 | 0.75 | 0.02629 | P > 0.05 | ns |
| 60.00 | 30.5 | 1.069 | P > 0.05 | ns |
| 120.0 | 7 | 0.2454 | P > 0.05 | ns |

| **Figure 3d** | | | | | |
| --- | --- | --- | --- | --- | --- |
| Bonferroni's Multiple Comparison Test | Mean Diff. | t | Significant? P < 0.05? | Summary | 95% CI of diff |
| WT, RD vs WT, FSD | -1.711 | 5.197 | Yes | *** | -2.637 to -0.7850 |
| WT, RD vs Txnip-KO, RD | 0.06333 | 0.1924 | No | ns | -0.8625 to 0.9892 |
| WT, RD vs Txnip-KO, FSD | -0.238 | 0.723 | No | ns | -1.164 to 0.6878 |
| WT, FSD vs Txnip-KO, RD | 1.774 | 5.389 | Yes | *** | 0.8483 to 2.700 |
| WT, FSD vs Txnip-KO, FSD | 1.473 | 4.474 | Yes | *** | 0.5470 to 2.399 |
| Txnip-KO, RD vs Txnip-KO, FSD | -0.3013 | 0.9153 | No | ns | -1.227 to 0.6245 |

| **Figure 3e** |  |  |  |  |  |
| --- | --- | --- | --- | --- | --- |
| Bonferroni's Multiple Comparison Test | Mean Diff. | t | Significant? P < 0.05? | Summary | 95% CI of diff |
| WT, RD vs WT, FSD | -2.083 | 9.246 | Yes | *** | -2.704 to -1.463 |
| WT, RD vs Txnip-KO, RD | 0.08333 | 0.3698 | No | ns | -0.5373 to 0.7040 |
| WT, RD vs Txnip-KO, FSD | -1.117 | 5.224 | Yes | *** | -1.705 to -0.5279 |
| WT, FSD vs Txnip-KO, RD | 2.167 | 9.616 | Yes | *** | 1.546 to 2.787 |
| WT, FSD vs Txnip-KO, FSD | 0.9667 | 4.522 | Yes | *** | 0.3779 to 1.555 |
| Txnip-KO, RD vs Txnip-KO, FSD | -1.2 | 5.614 | Yes | *** | -1.789 to -0.6112 |
