# Supplementary material for: Diabetes regulates fructose absorption through thioredoxin-interacting protein
Source: eLife. 2016 Oct 11;5:e18313. doi: 10.7554/eLife.18313 (PMC5059142; doi:10.7554/eLife.18313)
Supplement: Figure 4—source data 1. — These tables represent the statistical analysis conducted on the raw data collected for Figure 4 using GraphPad Prism 5. DOI: http://dx.doi.org/10.7554/eLife.18313.013 [file elife-18313-fig4-data1.docx]

**Figure 4-source data 1 | Statistical Analysis for Figure 4**

| **Figure 4a** | | | | | |
| --- | --- | --- | --- | --- | --- |
| Bonferroni's Multiple Comparison Test | Mean Diff. | t | Significant? P < 0.05? | Summary | 95% CI of diff |
| WT, RD vs WT, FSD | -1.484 | 2.79 | Yes | * | -2.960 to -0.007506 |
| WT, RD vs Txnip-KO, RD | -0.2684 | 0.4812 | No | ns | -1.817 to 1.280 |
| WT, RD vs Txnip-KO, FSD | -1.902 | 3.411 | Yes | ** | -3.451 to -0.3543 |
| WT, FSD vs Txnip-KO, RD | 1.215 | 2.179 | No | ns | -0.3329 to 2.763 |
| WT, FSD vs Txnip-KO, FSD | -0.4188 | 0.7509 | No | ns | -1.967 to 1.129 |
| Txnip-KO, RD vs Txnip-KO, FSD | -1.634 | 2.805 | Yes | * | -3.251 to -0.01705 |

| **Figure 4b** | | | | | |
| --- | --- | --- | --- | --- | --- |
| Bonferroni's Multiple Comparison Test | Mean Diff. | t | Significant? P < 0.05? | Summary | 95% CI of diff |
| WT, RD vs WT, FSD | -2.584 | 3.217 | Yes | * | -4.814 to -0.3542 |
| WT, RD vs Txnip-KO, RD | 0.1586 | 0.1882 | No | ns | -2.180 to 2.497 |
| WT, RD vs Txnip-KO, FSD | -2.405 | 2.855 | Yes | * | -4.743 to -0.06633 |
| WT, FSD vs Txnip-KO, RD | 2.743 | 3.255 | Yes | * | 0.4040 to 5.081 |
| WT, FSD vs Txnip-KO, FSD | 0.1791 | 0.2125 | No | ns | -2.160 to 2.518 |
| Txnip-KO, RD vs Txnip-KO, FSD | -2.564 | 2.913 | Yes | * | -5.006 to -0.1209 |

| **Figure 4c** | | | | | |
| --- | --- | --- | --- | --- | --- |
| Bonferroni's Multiple Comparison Test | Mean Diff. | t | Significant? P < 0.05? | Summary | 95% CI of diff |
| WT, RD vs WT, FSD | -1.274 | 3.794 | Yes | ** | -2.205 to -0.3417 |
| WT, RD vs Txnip-KO, RD | 1.109 | 3.15 | Yes | * | 0.1316 to 2.086 |
| WT, RD vs Txnip-KO, FSD | 1.048 | 2.976 | Yes | * | 0.07062 to 2.025 |
| WT, FSD vs Txnip-KO, RD | 2.383 | 6.767 | Yes | *** | 1.405 to 3.360 |
| WT, FSD vs Txnip-KO, FSD | 2.322 | 6.593 | Yes | *** | 1.344 to 3.299 |
| Txnip-KO, RD vs Txnip-KO, FSD | -0.06103 | 0.1659 | No | ns | -1.082 to 0.9598 |

| **Figure 4d** | |
| --- | --- |
| Table Analyzed | GLUT2 |
| Column A | RD |
| vs | vs |
| Column B | FSD |
|  |  |
| Unpaired t test |  |
| P value | 0.0114 |
| P value summary | * |
| Are means signif. different? (P < 0.05) | Yes |
| One- or two-tailed P value? | Two-tailed |
| t, df | t=2.984 df=12 |
|  |  |
| How big is the difference? |  |
| Mean ± SEM of column A | 10.24 ± 1.687 N=6 |
| Mean ± SEM of column B | 22.44 ± 3.726 N=6 |
| Difference between means | -12.20 ± 4.090 |
| 95% confidence interval | -21.12 to -3.291 |
| R square | 0.4259 |

| **Figure 4e** | |
| --- | --- |
| Table Analyzed | GLUT5 |
| Column A | RD |
| vs | vs |
| Column B | FSD |
|  |  |
| Unpaired t test |  |
| P value | 0.0255 |
| P value summary | * |
| Are means signif. different? (P < 0.05) | Yes |
| One- or two-tailed P value? | Two-tailed |
| t, df | t=2.582 df=11 |
|  |  |
| How big is the difference? |  |
| Mean ± SEM of column A | 4.299 ± 1.288 N=6 |
| Mean ± SEM of column B | 10.25 ± 1.820 N=6 |
| Difference between means | -5.953 ± 2.306 |
| 95% confidence interval | -11.03 to -0.8776 |
| R square | 0.3773 |
