# Supplementary material for: Diabetes regulates fructose absorption through thioredoxin-interacting protein
Source: eLife. 2016 Oct 11;5:e18313. doi: 10.7554/eLife.18313 (PMC5059142; doi:10.7554/eLife.18313)
Supplement: Figure 5—source data 1. — These tables represent the statistical analysis conducted on the raw data collected for Figure 5 using GraphPad Prism 5. DOI: http://dx.doi.org/10.7554/eLife.18313.017 [file elife-18313-fig5-data1.docx]

**Figure 5-source data 1 | Statistical Analysis for Figure 5**

| **Figure 5a** | | | | | |
| --- | --- | --- | --- | --- | --- |
| Bonferroni's Multiple Comparison Test | Mean Diff. | t | Significant? P < 0.05? | Summary | 95% CI of diff |
| WT vs WT + STZ | -3.334 | 8.346 | Yes | *** | -4.469 to -2.200 |
| WT vs Txnip-KO | 0.8369 | 2.095 | No | ns | -0.2973 to 1.971 |
| WT vs Txnip-KO + STZ | 0.8015 | 2.006 | No | ns | -0.3327 to 1.936 |
| WT + STZ vs Txnip-KO | 4.171 | 10.44 | Yes | *** | 3.037 to 5.305 |
| WT + STZ vs Txnip-KO + STZ | 4.136 | 10.35 | Yes | *** | 3.002 to 5.270 |
| Txnip-KO vs Txnip-KO + STZ | -0.03536 | 0.08852 | No | ns | -1.170 to 1.099 |

| **Figure 5b** | | | | | |
| --- | --- | --- | --- | --- | --- |
| Bonferroni posttests |  |  |  |  |  |
|  |  |  |  |  |  |
| WT control vs WT STZ |  |  |  |  |  |
| Column Factor | WT control | WT STZ | Difference | 95% CI of diff. |  |
| 0.0 | 0 | 0 | 0 | -21472 to 21472 |  |
| 5.000 | 19711 | 28891 | 9180 | -12293 to 30652 |  |
| 10.00 | 38198 | 68284 | 30086 | 8613 to 51558 |  |
| 20.00 | 28022 | 47253 | 19230 | -2242 to 40703 |  |
| 30.00 | 9729 | 14898 | 5168 | -16304 to 26640 |  |
| 60.00 | 9152 | 13460 | 4308 | -17164 to 25780 |  |
|  |  |  |  |  |  |
| Column Factor | Difference | t | P value | Summary |  |
| 0.0 | 0 | 0 | P > 0.05 | ns |  |
| 5.000 | 9180 | 1.422 | P > 0.05 | ns |  |
| 10.00 | 30086 | 4.66 | P<0.001 | *** |  |
| 20.00 | 19230 | 2.979 | P < 0.05 | * |  |
| 30.00 | 5168 | 0.8006 | P > 0.05 | ns |  |
| 60.00 | 4308 | 0.6673 | P > 0.05 | ns |  |
|  |  |  |  |  |  |
| WT control vs KO control |  |  |  |  |  |
| Column Factor | WT control | KO control | Difference | 95% CI of diff. |  |
| 0.0 | 0 | 0 | 0 | -21472 to 21472 |  |
| 5.000 | 19711 | 11614 | -8097 | -29570 to 13375 |  |
| 10.00 | 38198 | 17335 | -20863 | -42336 to 609.0 |  |
| 20.00 | 28022 | 15799 | -12223 | -33695 to 9249 |  |
| 30.00 | 9729 | 4932 | -4797 | -26270 to 16675 |  |
| 60.00 | 9152 | 6135 | -3017 | -24489 to 18455 |  |
|  |  |  |  |  |  |
| Column Factor | Difference | t | P value | Summary |  |
| 0.0 | 0 | 0 | P > 0.05 | ns |  |
| 5.000 | -8097 | 1.254 | P > 0.05 | ns |  |
| 10.00 | -20863 | 3.232 | P < 0.05 | * |  |
| 20.00 | -12223 | 1.893 | P > 0.05 | ns |  |
| 30.00 | -4797 | 0.7431 | P > 0.05 | ns |  |
| 60.00 | -3017 | 0.4673 | P > 0.05 | ns |  |
|  |  |  |  |  |  |
| WT control vs KO STZ |  |  |  |  |  |
| Column Factor | WT control | KO STZ | Difference | 95% CI of diff. |  |
| 0.0 | 0 | 0 | 0 | -21472 to 21472 |  |
| 5.000 | 19711 | 13335 | -6376 | -27848 to 15096 |  |
| 10.00 | 38198 | 25041 | -13158 | -34630 to 8315 |  |
| 20.00 | 28022 | 21173 | -6849 | -28322 to 14623 |  |
| 30.00 | 9729 | 8825 | -904 | -22376 to 20568 |  |
| 60.00 | 9152 | 8919 | -233.3 | -21706 to 21239 |  |
|  |  |  |  |  |  |
| Column Factor | Difference | t | P value | Summary |  |
| 0.0 | 0 | 0 | P > 0.05 | ns |  |
| 5.000 | -6376 | 0.9876 | P > 0.05 | ns |  |
| 10.00 | -13158 | 2.038 | P > 0.05 | ns |  |
| 20.00 | -6849 | 1.061 | P > 0.05 | ns |  |
| 30.00 | -904 | 0.14 | P > 0.05 | ns |  |
| 60.00 | -233.3 | 0.03613 | P > 0.05 | ns |  |
|  |  |  |  |  |  |
| WT STZ vs KO control |  |  |  |  |  |
| Column Factor | WT STZ | KO control | Difference | 95% CI of diff. |  |
| 0.0 | 0 | 0 | 0 | -21472 to 21472 |  |
| 5.000 | 28891 | 11614 | -17277 | -38749 to 4195 |  |
| 10.00 | 68284 | 17335 | -50949 | -72421 to -29477 |  |
| 20.00 | 47253 | 15799 | -31453 | -52926 to -9981 |  |
| 30.00 | 14898 | 4932 | -9966 | -31438 to 11507 |  |
| 60.00 | 13460 | 6135 | -7325 | -28797 to 14148 |  |
|  |  |  |  |  |  |
| Column Factor | Difference | t | P value | Summary |  |
| 0.0 | 0 | 0 | P > 0.05 | ns |  |
| 5.000 | -17277 | 2.676 | P > 0.05 | ns |  |
| 10.00 | -50949 | 7.892 | P<0.001 | *** |  |
| 20.00 | -31453 | 4.872 | P<0.001 | *** |  |
| 30.00 | -9966 | 1.544 | P > 0.05 | ns |  |
| 60.00 | -7325 | 1.135 | P > 0.05 | ns |  |
|  |  |  |  |  |  |
| WT STZ vs KO STZ |  |  |  |  |  |
| Column Factor | WT STZ | KO STZ | Difference | 95% CI of diff. |  |
| 0.0 | 0 | 0 | 0 | -21472 to 21472 |  |
| 5.000 | 28891 | 13335 | -15556 | -37028 to 5917 |  |
| 10.00 | 68284 | 25041 | -43244 | -64716 to -21771 |  |
| 20.00 | 47253 | 21173 | -26080 | -47552 to -4608 |  |
| 30.00 | 14898 | 8825 | -6072 | -27545 to 15400 |  |
| 60.00 | 13460 | 8919 | -4541 | -26013 to 16931 |  |
|  |  |  |  |  |  |
| Column Factor | Difference | t | P value | Summary |  |
| 0.0 | 0 | 0 | P > 0.05 | ns |  |
| 5.000 | -15556 | 2.41 | P > 0.05 | ns |  |
| 10.00 | -43244 | 6.698 | P<0.001 | *** |  |
| 20.00 | -26080 | 4.04 | P<0.001 | *** |  |
| 30.00 | -6072 | 0.9406 | P > 0.05 | ns |  |
| 60.00 | -4541 | 0.7034 | P > 0.05 | ns |  |
|  |  |  |  |  |  |
| KO control vs KO STZ |  |  |  |  |  |
| Column Factor | KO control | KO STZ | Difference | 95% CI of diff. |  |
| 0.0 | 0 | 0 | 0 | -21472 to 21472 |  |
| 5.000 | 11614 | 13335 | 1721 | -19751 to 23194 |  |
| 10.00 | 17335 | 25041 | 7706 | -13767 to 29178 |  |
| 20.00 | 15799 | 21173 | 5373 | -16099 to 26846 |  |
| 30.00 | 4932 | 8825 | 3893 | -17579 to 25366 |  |
| 60.00 | 6135 | 8919 | 2784 | -18689 to 24256 |  |
|  |  |  |  |  |  |
| Column Factor | Difference | t | P value | Summary |  |
| 0.0 | 0 | 0 | P > 0.05 | ns |  |
| 5.000 | 1721 | 0.2666 | P > 0.05 | ns |  |
| 10.00 | 7706 | 1.194 | P > 0.05 | ns |  |
| 20.00 | 5373 | 0.8324 | P > 0.05 | ns |  |
| 30.00 | 3893 | 0.6031 | P > 0.05 | ns |  |
| 60.00 | 2784 | 0.4312 | P > 0.05 | ns |  |
|  |  |  |  |  |  |
| Bonferroni's Multiple Comparison Test | Mean Diff. | t | Significant? P < 0.05? | Summary | 95% CI of diff |
| WT control vs WT STZ | -605987 | 3.293 | Yes | * | -1.186e+006 to -25798 |
| WT control vs KO control | 373245 | 2.028 | No | ns | -206943 to 953434 |
| WT control vs KO STZ | 322864 | 1.754 | No | ns | -257325 to 903052 |
| WT STZ vs KO control | 979232 | 5.321 | Yes | ** | 399043 to 1.559e+006 |
| WT STZ vs KO STZ | 928850 | 5.047 | Yes | ** | 348662 to 1.509e+006 |
| KO control vs KO STZ | -50382 | 0.2738 | No | ns | -630570 to 529807 |

| **Figure 5c** | | | | | |
| --- | --- | --- | --- | --- | --- |
| Bonferroni posttests |  |  |  |  |  |
|  |  |  |  |  |  |
| WT control vs WT STZ |  |  |  |  |  |
| Column Factor | WT control | WT STZ | Difference | 95% CI of diff. |  |
| 0.0 | 0 | 0 | 0 | -2211 to 2211 |  |
| 5.000 | 3461 | 5176 | 1715 | -496.0 to 3927 |  |
| 10.00 | 5149 | 8688 | 3539 | 1328 to 5751 |  |
| 20.00 | 6359 | 9256 | 2897 | 685.7 to 5109 |  |
| 30.00 | 4440 | 6141 | 1700 | -511.0 to 3912 |  |
| 60.00 | 3114 | 2911 | -203.2 | -2415 to 2008 |  |
|  |  |  |  |  |  |
| Column Factor | Difference | t | P value | Summary |  |
| 0.0 | 0 | 0 | P > 0.05 | ns |  |
| 5.000 | 1715 | 2.58 | P > 0.05 | ns |  |
| 10.00 | 3539 | 5.323 | P<0.001 | *** |  |
| 20.00 | 2897 | 4.357 | P<0.001 | *** |  |
| 30.00 | 1700 | 2.558 | P > 0.05 | ns |  |
| 60.00 | -203.2 | 0.3056 | P > 0.05 | ns |  |
|  |  |  |  |  |  |
| WT control vs KO control |  |  |  |  |  |
| Column Factor | WT control | KO control | Difference | 95% CI of diff. |  |
| 0.0 | 0 | 0 | 0 | -2211 to 2211 |  |
| 5.000 | 3461 | 2586 | -874.3 | -3086 to 1337 |  |
| 10.00 | 5149 | 4266 | -883 | -3094 to 1328 |  |
| 20.00 | 6359 | 4199 | -2160 | -4371 to 51.64 |  |
| 30.00 | 4440 | 2501 | -1939 | -4151 to 272.1 |  |
| 60.00 | 3114 | 2667 | -447.5 | -2659 to 1764 |  |
|  |  |  |  |  |  |
| Column Factor | Difference | t | P value | Summary |  |
| 0.0 | 0 | 0 | P > 0.05 | ns |  |
| 5.000 | -874.3 | 1.315 | P > 0.05 | ns |  |
| 10.00 | -883 | 1.328 | P > 0.05 | ns |  |
| 20.00 | -2160 | 3.248 | P < 0.05 | * |  |
| 30.00 | -1939 | 2.917 | P < 0.05 | * |  |
| 60.00 | -447.5 | 0.673 | P > 0.05 | ns |  |
|  |  |  |  |  |  |
| WT control vs KO STZ |  |  |  |  |  |
| Column Factor | WT control | KO STZ | Difference | 95% CI of diff. |  |
| 0.0 | 0 | 0 | 0 | -2211 to 2211 |  |
| 5.000 | 3461 | 1993 | -1467 | -3679 to 744.3 |  |
| 10.00 | 5149 | 4697 | -452.3 | -2664 to 1759 |  |
| 20.00 | 6359 | 4765 | -1594 | -3805 to 617.7 |  |
| 30.00 | 4440 | 3775 | -665.6 | -2877 to 1546 |  |
| 60.00 | 3114 | 3422 | 307.2 | -1904 to 2519 |  |
|  |  |  |  |  |  |
| Column Factor | Difference | t | P value | Summary |  |
| 0.0 | 0 | 0 | P > 0.05 | ns |  |
| 5.000 | -1467 | 2.207 | P > 0.05 | ns |  |
| 10.00 | -452.3 | 0.6802 | P > 0.05 | ns |  |
| 20.00 | -1594 | 2.397 | P > 0.05 | ns |  |
| 30.00 | -665.6 | 1.001 | P > 0.05 | ns |  |
| 60.00 | 307.2 | 0.4621 | P > 0.05 | ns |  |
|  |  |  |  |  |  |
| WT STZ vs KO control |  |  |  |  |  |
| Column Factor | WT STZ | KO control | Difference | 95% CI of diff. |  |
| 0.0 | 0 | 0 | 0 | -2211 to 2211 |  |
| 5.000 | 5176 | 2586 | -2590 | -4801 to -378.2 |  |
| 10.00 | 8688 | 4266 | -4422 | -6634 to -2211 |  |
| 20.00 | 9256 | 4199 | -5057 | -7268 to -2846 |  |
| 30.00 | 6141 | 2501 | -3640 | -5851 to -1428 |  |
| 60.00 | 2911 | 2667 | -244.3 | -2456 to 1967 |  |
|  |  |  |  |  |  |
| Column Factor | Difference | t | P value | Summary |  |
| 0.0 | 0 | 0 | P > 0.05 | ns |  |
| 5.000 | -2590 | 3.895 | P<0.01 | ** |  |
| 10.00 | -4422 | 6.651 | P<0.001 | *** |  |
| 20.00 | -5057 | 7.606 | P<0.001 | *** |  |
| 30.00 | -3640 | 5.474 | P<0.001 | *** |  |
| 60.00 | -244.3 | 0.3674 | P > 0.05 | ns |  |
|  |  |  |  |  |  |
| WT STZ vs KO STZ |  |  |  |  |  |
| Column Factor | WT STZ | KO STZ | Difference | 95% CI of diff. |  |
| 0.0 | 0 | 0 | 0 | -2211 to 2211 |  |
| 5.000 | 5176 | 1993 | -3183 | -5394 to -971.1 |  |
| 10.00 | 8688 | 4697 | -3992 | -6203 to -1780 |  |
| 20.00 | 9256 | 4765 | -4491 | -6702 to -2279 |  |
| 30.00 | 6141 | 3775 | -2366 | -4577 to -154.6 |  |
| 60.00 | 2911 | 3422 | 510.4 | -1701 to 2722 |  |
|  |  |  |  |  |  |
| Column Factor | Difference | t | P value | Summary |  |
| 0.0 | 0 | 0 | P > 0.05 | ns |  |
| 5.000 | -3183 | 4.787 | P<0.001 | *** |  |
| 10.00 | -3992 | 6.003 | P<0.001 | *** |  |
| 20.00 | -4491 | 6.754 | P<0.001 | *** |  |
| 30.00 | -2366 | 3.559 | P<0.01 | ** |  |
| 60.00 | 510.4 | 0.7676 | P > 0.05 | ns |  |
|  |  |  |  |  |  |
| KO control vs KO STZ |  |  |  |  |  |
| Column Factor | KO control | KO STZ | Difference | 95% CI of diff. |  |
| 0.0 | 0 | 0 | 0 | -2211 to 2211 |  |
| 5.000 | 2586 | 1993 | -592.9 | -2804 to 1619 |  |
| 10.00 | 4266 | 4697 | 430.7 | -1781 to 2642 |  |
| 20.00 | 4199 | 4765 | 566.1 | -1645 to 2778 |  |
| 30.00 | 2501 | 3775 | 1274 | -937.7 to 3485 |  |
| 60.00 | 2667 | 3422 | 754.7 | -1457 to 2966 |  |
|  |  |  |  |  |  |
| Column Factor | Difference | t | P value | Summary |  |
| 0.0 | 0 | 0 | P > 0.05 | ns |  |
| 5.000 | -592.9 | 0.8917 | P > 0.05 | ns |  |
| 10.00 | 430.7 | 0.6478 | P > 0.05 | ns |  |
| 20.00 | 566.1 | 0.8514 | P > 0.05 | ns |  |
| 30.00 | 1274 | 1.916 | P > 0.05 | ns |  |
| 60.00 | 754.7 | 1.135 | P > 0.05 | ns |  |
|  |  |  |  |  |  |
| Bonferroni's Multiple Comparison Test | Mean Diff. | t | Significant? P < 0.05? | Summary | 95% CI of diff |
| WT vs WT, STZ | -86553 | 4.421 | Yes | ** | -148281 to -24824 |
| WT vs Txnip-KO | 72053 | 3.68 | Yes | * | 10325 to 133782 |
| WT vs Txnip-KO, STZ | 32040 | 1.636 | No | ns | -29688 to 93769 |
| WT, STZ vs Txnip-KO | 158606 | 8.101 | Yes | *** | 96878 to 220334 |
| WT, STZ vs Txnip-KO, STZ | 118593 | 6.057 | Yes | *** | 56865 to 180321 |
| Txnip-KO vs Txnip-KO, STZ | -40013 | 2.044 | No | ns | -101741 to 21715 |
