# Supplementary material for: Diabetes regulates fructose absorption through thioredoxin-interacting protein
Source: eLife. 2016 Oct 11;5:e18313. doi: 10.7554/eLife.18313 (PMC5059142; doi:10.7554/eLife.18313)
Supplement: Figure 5—source data 2. — This table represents the statistical analysis conducted on the raw data collected for Figure 5—figure supplement 1 using GraphPad Prism 5. DOI: http://dx.doi.org/10.7554/eLife.18313.018 [file elife-18313-fig5-data2.docx]

**Figure 5-source data 2 | Statistical Analysis for Figure 5-figure supplement 1**

| Bonferroni's Multiple Comparison Test | Mean Diff. | t | Significant? P < 0.05? | Summary | 95% CI of diff |
| --- | --- | --- | --- | --- | --- |
| WT vs WT STZ | -281.4 | 7.806 | Yes | *** | -382.0 to -180.8 |
| WT vs KO | 69.4 | 1.925 | No | ns | -31.24 to 170.0 |
| WT vs KO STZ | -133.3 | 3.698 | Yes | ** | -233.9 to -32.66 |
| WT STZ vs KO | 350.8 | 9.732 | Yes | *** | 250.2 to 451.4 |
| WT STZ vs KO STZ | 148.1 | 4.109 | Yes | ** | 47.46 to 248.7 |
| KO vs KO STZ | -202.7 | 5.623 | Yes | *** | -303.3 to -102.1 |
