# Supplementary material for: Diabetes regulates fructose absorption through thioredoxin-interacting protein
Source: eLife. 2016 Oct 11;5:e18313. doi: 10.7554/eLife.18313 (PMC5059142; doi:10.7554/eLife.18313)
Supplement: Figure 5—source data 3. — DOI: http://dx.doi.org/10.7554/eLife.18313.019 [file elife-18313-fig5-data3.docx]

**Figure 5-source data 3 | Statistical Analysis for Figure 5-figure supplement 2**

| Table Analyzed | Small Intestine |
| --- | --- |
| Column A | WT control |
| vs | vs |
| Column B | WT STZ |
|  |  |
| Unpaired t test |  |
| P value | 0.0256 |
| P value summary | * |
| Are means signif. different? (P < 0.05) | Yes |
| One- or two-tailed P value? | Two-tailed |
| t, df | t=2.951 df=6 |
|  |  |
| How big is the difference? |  |
| Mean ± SEM of column A | 0.5672 ± 0.1496 N=4 |
| Mean ± SEM of column B | 1.391 ± 0.2357 N=4 |
| Difference between means | -0.8238 ± 0.2792 |
| 95% confidence interval | -1.507 to -0.1406 |
| R square | 0.592 |
