# Supplementary material for: Diabetes regulates fructose absorption through thioredoxin-interacting protein
Source: eLife. 2016 Oct 11;5:e18313. doi: 10.7554/eLife.18313 (PMC5059142; doi:10.7554/eLife.18313)
Supplement: Supplementary file 1. — DOI: http://dx.doi.org/10.7554/eLife.18313.022 [file elife-18313-supp1.docx]

**Supplementary file 1 | Primers used for quantitative RT-PCR analysis**

| **Gene** | **Forward/Reverse** | **Sequence (5’ 🡪 3’)** |
| --- | --- | --- |
| TBP | Forward | GCAGTGCCCAGCATCACTAT |
| TBP | Reverse | GCCCTGAGCATAAGGTGGAA |
| Txnip | Forward | CCGGACGGGTAATAGTGGAAG |
| Txnip | Reverse | CTCGTTCTCACCTGCTGTAG |
| GLUT2 | Forward | TCCCTTGGTTCATGGTTGCT |
| GLUT2 | Reverse | GGAAGTCCGCAATGTACTGGA |
| GLUT5 | Forward | CCCACCTACCCTGTTGAGAA |
| GLUT5 | Reverse | GGCTCTTCAATTCGGGGGC |
